# Microphysiological Solid Tumor Models in Hydrogel Beads for CAR T Cell Immunotherapy Evaluation

**DOI:** 10.1002/advs.202508267

**Published:** 2025-07-24

**Authors:** Xuan Peng, Željko Janićijević, Liliana R. Loureiro, Lydia Hoffmann, Poh Soo Lee, Isli Cela, Benjamin Kruppke, Alexandra Kegler, Anja Feldmann, Ielizaveta Gorodetska, Anja Madleine Markl, Anna Dubrovska, Anne Kathrin Offermann, Michael Bachmann, Larysa Baraban

**Affiliations:** ^1^ Helmholtz‐Zentrum Dresden‐Rossendorf Institute of Radiopharmaceutical Cancer Research 01328 Dresden Germany; ^2^ Faculty of Medicine and University Hospital Carl Gustav Carus Technische Universität Dresden 01307 Dresden Germany; ^3^ Max Bergmann Center of Biomaterials and Institute of Materials Science Technische Universität Dresden 01069 Dresden Germany; ^4^ OncoRay – National Center for Radiation Research in Oncology Faculty of Medicine and University Hospital Carl Gustav Carus Dresden University of Technology Helmholtz‐Zentrum Dresden‐Rossendorf Institute of Radiooncology 28414 Dresden Germany; ^5^ Gerhard Domagk Institute of Pathology University Hospital Münster 48149 Münster Germany; ^6^ National Center for Tumor Diseases (NCT) 01307 Dresden Germany; ^7^ German Cancer Research Center (DKFZ) 69120 Heidelberg Germany; ^8^ German Cancer Consortium (DKTK) 01307 Dresden Germany

**Keywords:** droplet microfluidics, fibroblast activation protein, immunotherapy, micrometastases, PEGDA hydrogel beads, tumor microenvironment

## Abstract

Micrometastases are challenging to resect surgically and to detect with in vivo imaging. Immunotherapy is highly anticipated to revolutionize their treatment, but its overall efficacy still remains limited for solid tumors. Here, a 3D micrometastases model is developed to mimic key microenvironmental cues, enabling in vitro evaluation of chimeric antigen receptor (CAR) T cell immunotherapy. Prostate cancer that preferentially metastasizes to, *e.g*., liver or bone marrow, is utilized as a model. Hydrogel beads with an elastic modulus matching those of soft organs are used to support long‐term culturing, immunostaining, and monitoring of the spheroids. As a biochemical cue, the impact of fibroblast activation protein (FAP), an emerging target in the tumor microenvironment, is investigated on prostate cancer spheroids and on the efficacy of CAR T cell therapy. The multi‐spheroid model consists of prostate stem cell antigen (PSCA)‐expressing prostate cancer cells and FAP‐producing fibrosarcoma cells in varying ratios. The morphological features of the model are compared to clinical histopathology and metastatic murine model samples. Finally, CAR T cell trials demonstrate successful chemoattraction and infiltration through the hydrogel matrix, with a dual‐targeting approach against FAP and PSCA antigens showing synergistic efficacy. This research provides invaluable insights for engineering 3D tumor models and modeling therapies targeting small metastatic or residual tumors, suggesting that co‐targeting may be a more effective strategy to unlock the tumor microenvironment's suppression.

## Graphical abstract

The complex physical and biochemical barriers of the tumor microenvironment are major triggers of immune exclusion. Here, a 3D micrometastasis model is developed to mimic key microenvironmental cues, enabling in vitro evaluation of chimeric antigen receptor (CAR) T cell immunotherapy.

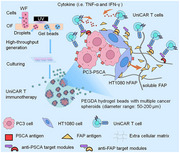

## 1 Introduction

The complexity of cancer and its escape mechanisms limit the therapeutic efficacy of cancer immunotherapy to ≈30–40% in clinical patients.^[^ ^1^, ^2^ ^]^ Cancer metastases – a leading cause of cancer‐related mortality – can compromise immunotherapy efficacy systemically in both patients and preclinical models.^[^ ^3^ ^]^ The tumor microenvironment (TME) at the structural and biochemical level is deemed responsible for the lower efficacy of immunotherapy in solid tumors, compared to, e.g., blood‐related malignancies.^[^ ^1^ ^]^ Rapid translation of effective treatments remains hindered by the lack of suitable cancer models recapitulating the inherent tumor heterogeneity, resistance to therapy, disease progression, and patient‐to‐patient variability.^[^ ^4^ ^]^ Recently, numerous efforts have been dedicated to preclinical in vitro cancer modeling,^[^ ^5^, ^6^ ^]^ particularly driven by advances in materials science and 3D cell culture technologies.^[^ ^7^, ^8^, ^9^ ^]^ Various biomaterials ranging from biologically derived polymers (such as matrigel,^[^ ^10^ ^]^ collagen^[^ ^11^ ^]^ and alginate^[^ ^12^, ^13^, ^14^ ^]^) to synthetic polymers (such as poly(ethylene glycol) diacrylate (PEGDA)^[^ ^15^, ^16^, ^17^ ^]^ and carboxymethyl cellulose^[^ ^18^ ^]^) have been studied to integrate the complex cellular assemblies, while only a minority of current 3D cancer models could be reconstructed in the existing biomaterial‐based matrices.^[^ ^4^ ^]^ These advancements have significantly enhanced our understanding of 3D cell culture models by providing an artificially created environment mimicking biological systems and enabling cells to proliferate or interact with their surroundings in 3D space.^[^ ^19^, ^20^ ^]^ However, mimicking key TME properties is still challenging, primarily due to: 1) the insufficient understanding of the role of physical cues (e.g., stiffness, viscosity, and viscoelasticity) that regulate tumor growth and invasion;^[^ ^21^, ^22^, ^23^ ^]^ 2) the inability to consistently incorporate key biomolecules that remodel the TME, influence the metastatic ability of cancer cells, and modulate immunotherapeutic responses, e.g., FAP known for its high expression in the tumor stroma, which can remodel the tumor microenvironment, promote tumor progress, and promote immune suppression (Table S1, Supporting Information); ^[^ ^24^, ^25^ ^]^ 3) the stromal cells (e.g., cancer‐associated fibroblasts (CAFs) and adipocytes) which play crucial roles in tumor metabolism, growth, and metastasis;^[^ ^26^ ^]^ 4) the distinct properties of certain cancer cell types, such as poor aggregation and limited stability in culture. For example, the human prostate cancer cells (PC3) reveal only a loose aggregation under 3D culture conditions and short‐term survival for a maximum of 10 days.^[^ ^27^, ^28^ ^]^

**Figure 1:**
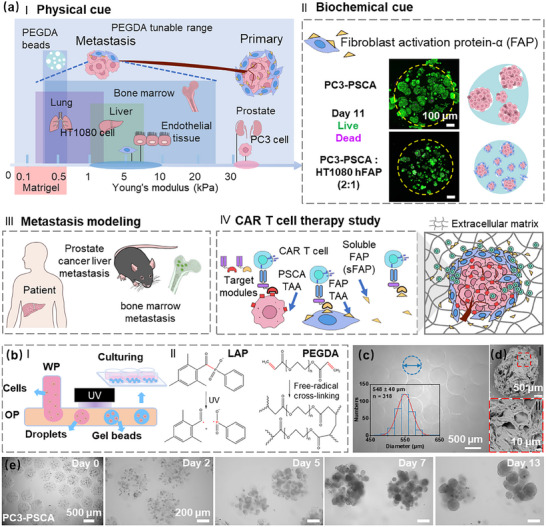
Conceptual illustration of the 3D tumor model and PEGDA hydrogel beads generation. a) Conceptual illustration of tumor tissue engineering for metastasis modeling and CAR T cell therapy studies. Key parameters within the TME include physical cues (I) such as elastic modulus (Young's modulus),^[^ ^37^ ^]^ and biochemical cues (II) ^[^ ^11^ ^]^ including cancer cells, stromal cells (such as cancer‐associated fibroblasts, which can express FAP), extracellular matrix (ECM), and soluble factors (such as cytokines and enzymes). In this study, prostate metastasis tumor models were created and compared with prostate cancer metastasis in vivo (III). A typical model, composed of PC3‐PSCA, HT1080 hFAP, and PEGDA hydrogel beads with adjustable stiffness, was used for a pilot CAR T cell therapy study to target the corresponding tumor‐associated antigens (TAA) on the cell membrane and soluble factors in the TME (IV). b) Schematic illustration of the T junction‐based microfluidic system coupled with the UV light source for the fabrication of PEGDA hydrogel microbeads (I); Mechanism of the PEGDA photopolymerization process (II).^[^ ^15^, ^38^ ^]^ c) A representative image of PEGDA hydrogel beads generated under the average UV intensity of 0.23 W cm^−2^; The used flow rate for water phase (WP) was 5.5 µL min^−1^ and for oil phase (OP) 110 µL min^−1^ (inset shows the distribution of generated PEGDA bead diameters indicating high monodispersity). d) Representative SEM images of freeze‐dried PEGDA hydrogel beads showcase the presence of macroscopic pores in the dry state. e) Representative images showing spheroid formation and proliferation in PEGDA hydrogel beads with PC3‐PSCA cells. PSCA: prostate stem cell antigen, FAP: fibroblast activation protein‐α, sFAP: soluble fibroblast activation protein. TMs: target modules, PEGDA: poly(ethylene glycol) diacrylate, LAP: lithium phenyl‐2,4,6‐trimethylbenzoylphosphinate.

To the best of our knowledge, most of the current 3D tumor models are focused on increasing biodiversity and mimicking tumor structures. Biomolecules, such as FAP, which contribute to tumor progression and offer a promising target for cancer therapy,^[^ ^29^, ^30^, ^31^ ^]^ were rarely considered when building 3D tumor models. The reasons described above restrict the possibilities of reaching sufficient tumor model maturation for use in immunotherapy (incl. CAR T cell therapy) investigations.^[^ ^32^, ^33^ ^]^ Thus, it is imperative to develop more adaptable 3D tumor models that offer the possibility to tune both physical (e.g., mechanical, **Figure** **1**a(I)) and chemical properties of the microenvironment, as well as demonstrate novel therapeutic approaches in vitro.

In this study, we explored both physical and biochemical cues impacting the microenvironment supporting the formation of spheroids using the hydrogel microbeads system that serves as an in vitro model for micrometastases. We utilized a prostate cancer case that preferentially metastasizes to softer organs compared to the primary tumor sites,^[^ ^6^, ^34^ ^]^ such as liver, lung, and bone marrow (Figure 1a (I&II)).^[^ ^35^ ^]^ Biocompatible PEGDA hydrogel beads with an elastic modulus similar to the soft organs are used here to mimic the extracellular matrix stiffness (Figure 1a (I)), enabling miniature, transparent, and reproducible microenvironments to facilitate long‐term cell culturing (up to 4 weeks), immunohistochemistry staining, and CAR‐T cell infiltration. The influence of FAP on the remodeling of tumor microenvironment was studied in a coculture system comprising PSCA‐modified prostate cancer cells (PC3‐PSCA) and the HT1080 expressing human FAP (HT1080 hFAP) cells (Figure 1a (II)). We further explored the structural and morphological similarities of our model to the histopathology in clinical patient and metastatic murine model samples (Figure 1a (III)). Finally, the 3D multi‐spheroid tumor models were also utilized to evaluate the infiltration of CAR T cells into the hydrogel matrix to reach the tumor regions and the efficacy of immunotherapy implemented using the Universal CAR (UniCAR) approach^[^ ^36^ ^]^ (Figure 1a (IV)). The results of our investigations based on the constructed microphysiological 3D tumor model indicate that: 1) physical cues significantly influence the formation of the 3D in vitro tumor model, and biochemical cues from FAP‐expressing cells and soluble FAP (sFAP) actively remodel the tumor microenvironment, together establishing the inhibitory physical and biochemical barriers; 2) this model closely mirrors the structural and morphological characteristics of mouse metastasis models and patient‐derived tumor samples, enhancing its relevance for evaluating cancer therapies; 3) CAR T cells overcome both physical and biochemical barriers to infiltrate the tumor regions, where a synergistic effect on cancer cell killing was demonstrated in the dual‐targeting CAR T cell therapy, underscoring its potential in overcoming the suppressive tumor microenvironment. This study holds promise for the facile design and engineering of in vitro 3D tumor models with tunable physical and biochemical elements of complex TME, showcasing a potential route to expedite the translation of CAR T cells into clinical immunotherapy procedures. Moreover, it provides valuable methodologies and insights for modeling therapies of small metastatic or residual tumor cells that cannot be easily resolved with traditional medical imaging modalities, and thus cannot be timely treated.

## 2 Results

### 2.1 Structure and Mechanical Properties of PEGDA Hydrogel Beads

To generate PEGDA hydrogel beads, we designed a droplet‐based microfluidic platform coupled to a 365‐nm UV lamp for photopolymerization (Figure 1b (I); Figure S1a, Supporting Information). Initially, the water phase (WP) containing 10% (w/v) of PEGDA monomers and 0.1% (w/v) of lithium phenyl‐2,4,6‐trimethylbenzoylphosphinate (LAP) photoinitiator dissolved in cell culture medium, and the mineral oil phase (OP), were separately injected into fluorinated ethylene propylene (FEP) tubes (outside diameter: 1.6 mm; inside diameter: 0.5 mm) with controlled flow rates using syringe pumps (Cetoni 210 base, q_WP_ = 5.5 µL min^−1^, q_OP_ = 110 µL min^−1^). The T‐junction facilitated the production of reproducible and monodisperse water‐in‐oil emulsion droplets that were further rapidly polymerized using UV irradiation (mean intensity: 0.23 W cm^−2^; exposure time: 2.5 s), resulting in hydrogel beads with an average diameter of 548 ± 40 µm (Figure 1c). The measurements and calculations of UV irradiation parameters are explained in “Preparation of PEGDA Hydrogel Beads and Size Measurement” and Note SI (Supporting Information), as well as in Figure S1b,c (Supporting Information). The polymerization reaction was achieved through the efficient absorption of light at 365 nm by the LAP photoinitiator, which resulted in the production of free radicals initiating the polymerization process (Figure 1b (II)),^[^ ^15^, ^38^ ^]^ entailing chain growth proceeding via covalent cross‐linking through the carbon double bonds of acrylate side groups terminating the PEGDA monomers.^[^ ^16^ ^]^ Cross‐linked PEGDA networks form hydrogels in aqueous media that in our case assume the shape of spherical beads with high monodispersity.

Fourier‐transform infrared spectroscopy analysis confirmed the successful formation of the cross‐linked PEGDA polymer network,^[^ ^16^, ^39^ ^]^ indicating no significant influence of the complex RPMI STINO cell culture medium on the final chemical composition of the hydrogel (Figure S2; Note SII, Supporting Information). The hydrogel beads represent a scaffold formed as a porous 3D cross‐linked polymer network (Figure 1d), providing good mechanical stability as well as sufficient space for cell culturing. Prostate cancer cells are sensitive to the mechanical properties of their microenvironment, and the elastic moduli of hydrogel beads play a crucial role in cell behavior. Mechanical analysis (MicroTester, Figure S3, Supporting Information, see ‘Elastic Modulus (Young's Modulus) Test’ for details) shows that the hydrogel beads exhibit Young´s moduli ranging from 300 to 600 Pa, increasing with the UV intensity used for bead generation (mean intensity: 0.23–0.35 W cm^−2^). The measured moduli range covers the values obtained for some major organs, such as liver, lung, and bone marrow (Figure 1a),^[^ ^34^, ^37^ ^]^ representing potential targets for prostate cancer metastases.

### 2.2 PEGDA Hydrogel Bead Tumor Models Mirroring In Vivo Metastases

**Figure 2:**
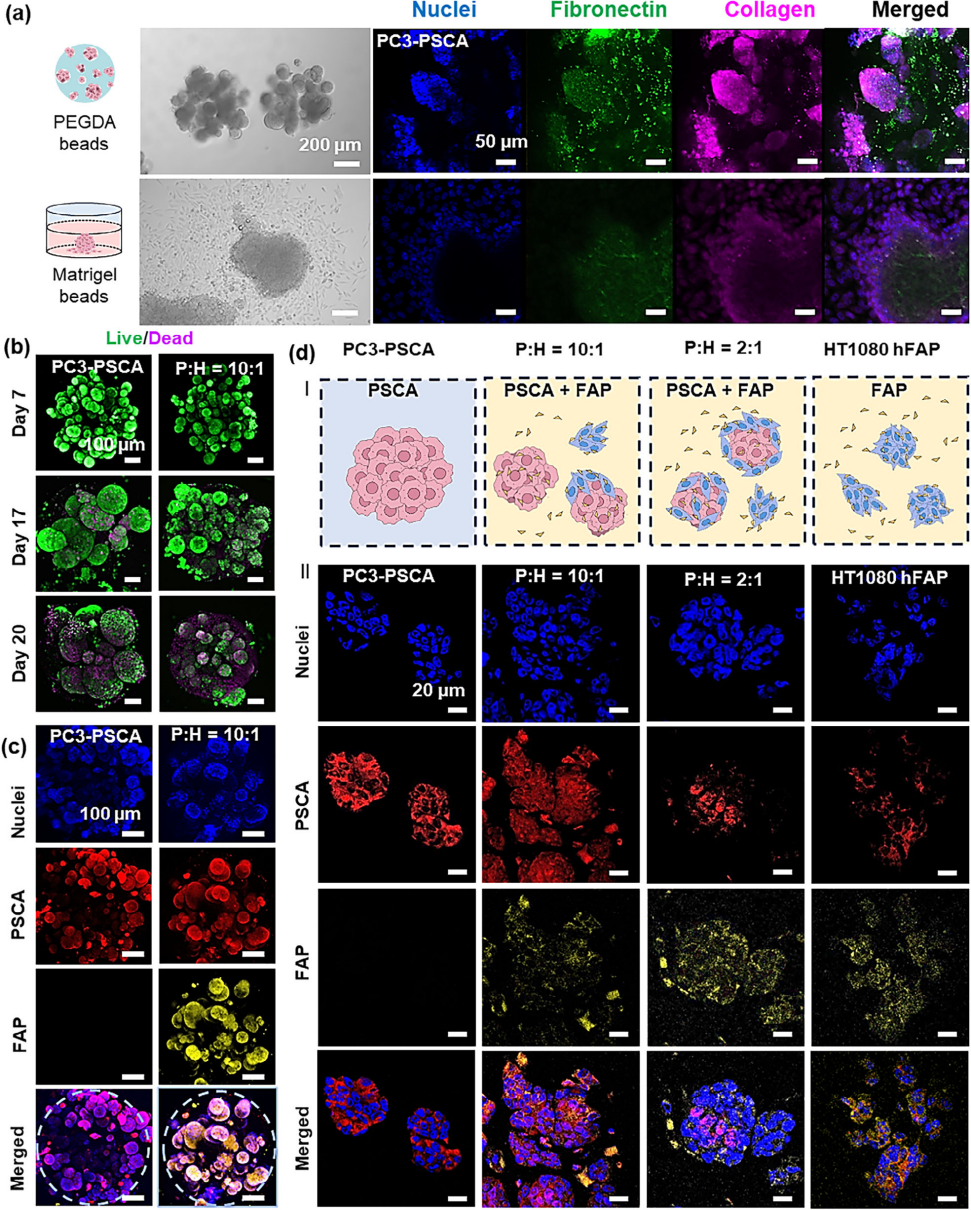
Different spheroids formed in PEGDA hydrogel beads. a) Cell aggregation, proliferation, and immunofluorescent staining of the extracellular matrix in PEGDA beads (day 7) and cell aggregation formed using Matrigel beads (day 3). Cyan: nuclei; green: fibronectin; magenta: collagen. b) Representative Live/Dead (green/magenta) staining images of PC3‐PSCA and P+H spheroids in PEGDA hydrogel beads. c) Immunostaining images of the structures of PC3‐PSCA and P+H spheroids in intact PEGDA hydrogel beads. d) Illustrations of different spheroid compositions and immunostaining images of spheroid sections on day 8 (cyan: nuclei; red: PSCA; yellow: FAP).

The PC3 spheroid formation in PEGDA was compared with several traditional methods (Note SV, Supporting Information), including U‐plate, bulk Matrigel, and micro Matrigel beads (Figures 1, 2; Figure S4, Supporting Information). It was observed that only in the PEGDA beads, the PC3 cells formed spheroids with a high level of fibronectin and collagen in the extracellular matrix. To investigate the influence of FAP‐expressing cells on spheroid formation, both mono‐cultured (PC3‐PSCA or HT1080 hFAP) and co‐cultured spheroids—where PC3‐PSCA and HT1080 hFAP were mixed at different ratios (P:H = 10:1, 2:1) —exhibited a robust growth in PEGDA hydrogel beads, sustaining culture for over 20 days. All spheroids exhibited solid cell aggregations. PC3‐PSCA and P+H spheroids (PC3‐PSCA to HT1080 hFAP ratios of 2:1 and 10:1), with the same initial total cell numbers, displayed similar growth trends within the first week (Figure 1e; Figure S5, Supporting Information). Thereafter, PC3 spheroids showed a faster increase in size compared to P+H spheroids. After two weeks, the average diameter of PC3‐PSCA spheroids reached 125 µm, compared to only 50 µm reached by P+H spheroids (for both P:H ratios: 2:1 and 10:1; Figure S6, Supporting Information). The smallest HT1080 hFAP spheroids had a highly heterogeneous morphology, making reliable size measurements difficult. Spheroids from different cultures can be maintained for ≈20 days (Figure 2b; Figure S7, Supporting Information). All groups indicated high viability (>90%) after the first week of culturing (Figure S7, Supporting Information). PC3‐PSCA spheroids exhibited viability above 90% up to ≈day 18. The PC3‐PSCA spheroid viability declined to ≈60% after 20 days, likely due to the lack of nutrients within the spheroids. P+H groups (ratios 10:1 and 2:1) showed fluctuations in viability during the second week, probably due to the nutrient competition between PC3‐PSCA and HT1080 hFAP cells. Spheroid viability in different groups showed significant differences on day 21, with higher cell viability associated with an increased HT1080 hFAP cell fraction. This effect is likely due to the sFAP that degrades the cell matrix and improves nutrient diffusion. Notably, the co‐culture with FAP cells leads to the overall smaller sizes of the spheroids that facilitate nutrient access to interior cells. HT1080 hFAP cells maintained consistent viability (80–95%) over three weeks without significant variation. The diameters of some PC3‐PSCA spheroids reached ≈300 µm, while the diameters of the P+H spheroids (PC3‐PSCA to HT1080 hFAP ratios of 2:1 and 10:1) were rarely larger than 100 µm. The HT1080 hFAP cells alone did not aggregate into regular spheroids. Spheroids tend to reach much smaller sizes for higher proportions of HT1080 hFAP cells in the initial cell culture, indicating that these cells hinder cell–cell aggregation. Such an effect could be attributed to the potential degradation of extracellular matrix components by FAP/sFAP which is expressed by HT1080 hFAP cells.^[^ ^24^, ^40^ ^]^

To gain insight into the structure of different spheroids, we conducted fluorescent immunostaining. PC3 cells abundantly express PSCA, while HT1080 cells intensely express FAP (Figure 2d). A lower level of PSCA was also observed in HT1080 hFAP cells (Figure 2d), lacking detectable PSCA on their surface (data not shown), following previous reports.^[^ ^41^ ^]^ FAP was observed exclusively on the surface of co‐cultured spheroids (Figure 2c). Immunostaining results of sections highlighted the heterogeneous nature of P+H spheroids and the presence of FAP or sFAP (see Figure 2d; Figure S9, Supporting Information). HT1080 hFAP was predominant in P+H (2:1) spheroids due to a higher abundance of HT1080 hFAP cells on the surface of spheroids compared to their interior. Interestingly, even when HT1080 hFAP accounted for only 10% of the initial culture, a significant amount of FAP was observed in the P+H (10:1) culturing experiments, thereby eventually indicating the formation of a “protective” layer for the tumor. Furthermore, immunofluorescent staining of the extracellular matrix confirmed its modification (Figure S9, Supporting Information). Our findings indicate that PEGDA hydrogel beads hosting multiple P+H spheroids provide the model in which hFAP cells serve as a stable source of cell‐associated and microenvironmental FAP/sFAP, a characteristic observed also in in vivo tumors.^[^ ^29^, ^30^, ^31^ ^]^

**Figure 3:**
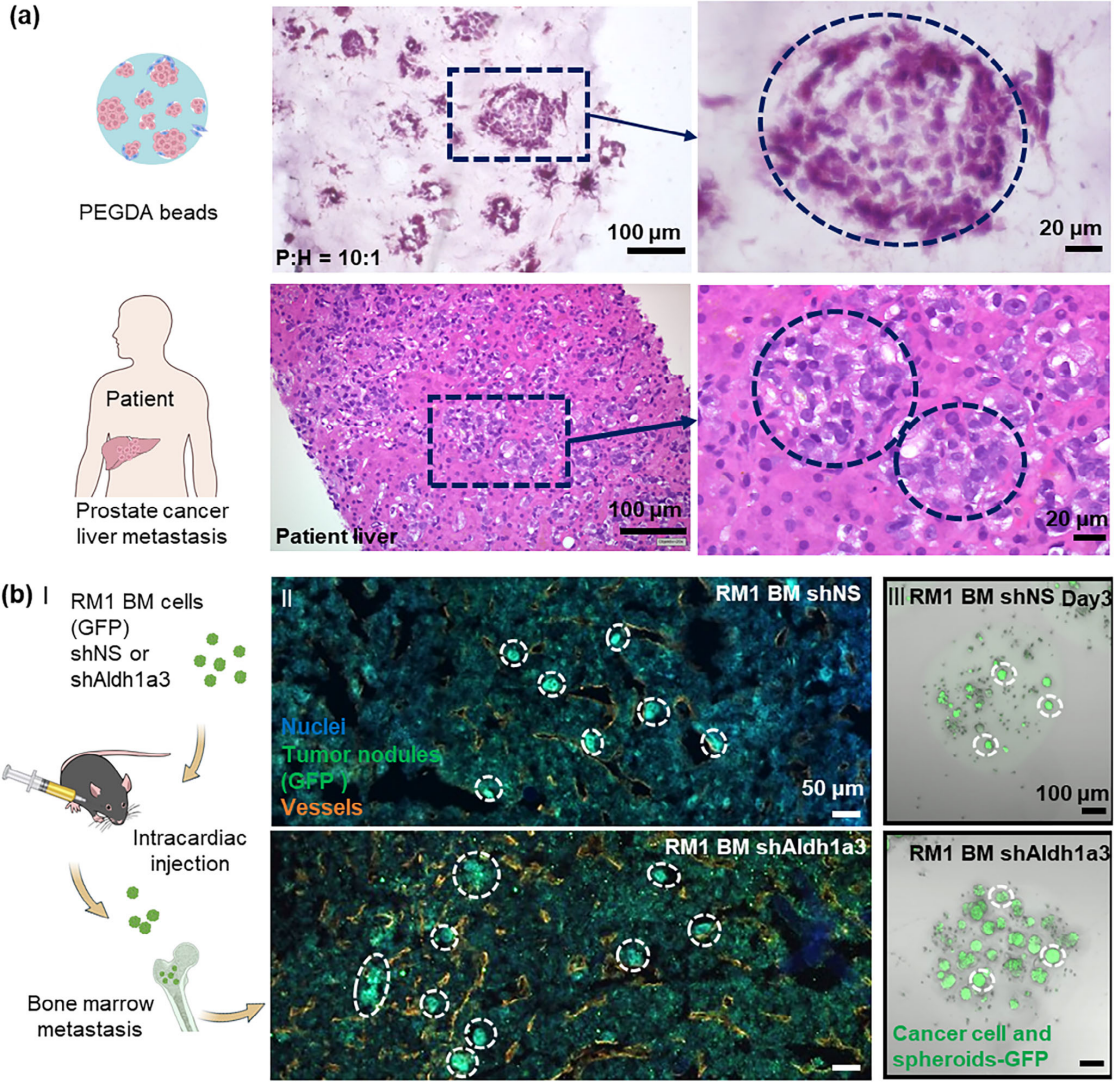
Similar to in vivo tissues. a) Comparison of H&E staining of P+H (10:1) spheroid sections after culturing for 8 days and prostate cancer liver metastasis sample from a patient. b) A schematic illustration of the experiment for the syngeneic mouse tumor model (I). 3 days after the injection of mouse prostate cancer RM1(BM) GFP+ cells, with or without Aldh1a3 depletion, tumor nodule formation was detected in the bones by immunofluorescence analysis (II). Proliferation and spheroid formation of RM1(BM) GFP+ cells in PEGDA beads (III). Cyan: nuclei; green: tumor cells and nodules (GFP); orange: vessels.

To prove that our system represents a comparable model that can be used to study metastatic prostate cancer, we further compare it to the a) samples obtained from the patient biopsy, i.e., with tissue microarrays from human prostate cancer metastases, and b) mouse metastasis models (see **Figure** **3**). First, sections of H&E‐stained P+H (10:1) spheroids indicated a structure similar to that observed in patient‐derived prostate cancer liver metastasis samples (Figure 3a). Further, we extend our comparative analysis toward mouse metastasis models. Here, the mouse prostate cancer bone‐metastatic cell line RM1(BM), with Aldh1a3 gene knockdown and previously reported high metastatic potential, was used.^[^ ^42^ ^]^ Intracardiac injection with RM1(BM) shAldh1a3 cells in immunocompetent mice exhibited a higher incidence of bone marrow metastases compared to those injected with the parental RM1(BM) cells treated with shNS (non‐silencing shRNA) (Figure 3bI&II). Consistent with our in vivo analysis (Figure 3bIII), RM1(BM) shAldh1a3 knockdown cells formed more spheroids within PEGDA beads than the original RM1(BM) shNS cell line. These findings reveal high morphological similarities to the images of our in vitro model, where both the original and metastatic cell line were grown inside of PEGDA beads.

### 2.3 CAR T Cell Migration and Infiltration

**Figure 4:**
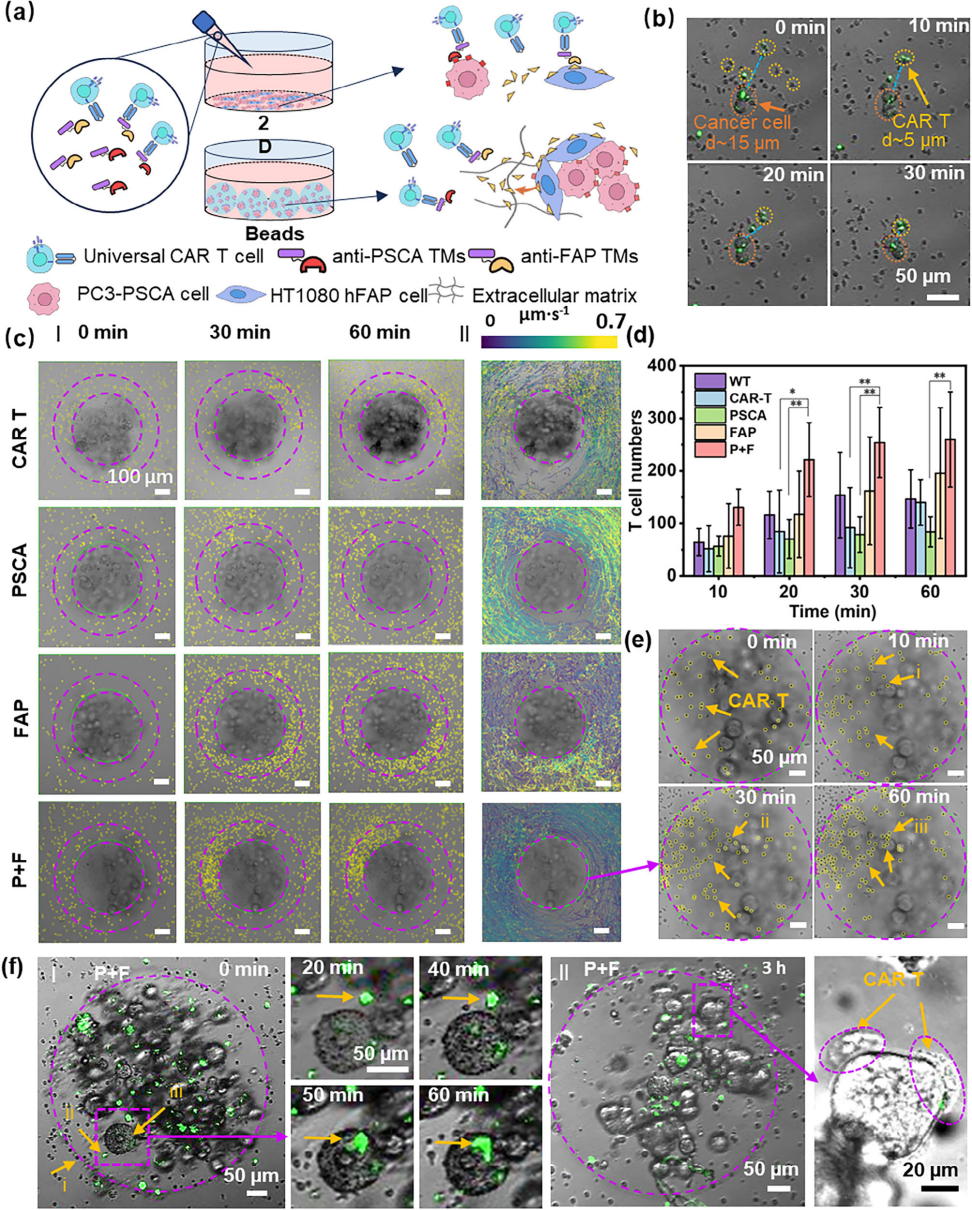
Differences in T cell approaches to cancer cells in PEGDA hydrogel beads with P+H (10:1) spheroids and under 2D culture conditions. a) A schematic illustration of CAR T cell targeting differences in PEGDA hydrogel beads and under 2D culture conditions (TMs: Target modules). b) CAR T cells approaching PC3 cancer cells in 2D culture. c) Representative images showing T cell distribution (small yellow circles) in wells under different culturing conditions (I) and tracking of T cell paths over 1 h (II). Each line represents an individual cell track. The color map indicates the instantaneous velocity values for T cells. CAR T: P+H spheroids with UniCAR T cells; PSCA: P+H spheroids with UniCAR T cells and anti‐PSCA TMs; FAP: P+H spheroids with UniCAR T cells and anti‐FAP TMs; P+F: P+H spheroids with UniCAR T cells, anti‐PSCA TMs, and anti‐FAP TMs. d) Average cell counts in ring areas designated in a(I) after culturing the spheroids and T cells for a certain period; the ring area has an inner diameter of 450 µm and an outer diameter of 800 µm, *n* = 3. e) Representative images of P+F group from Figure 3a(II) showing T cell distribution inside the hydrogel bead. f) (I) Representative time sequence of fluorescent micrographs illustrating the T cells approaching and contacting the spheroid within a PEGDA hydrogel bead; (II) Representative images of coordinated T cell accumulation on the surface of a spheroid after 3 h of culturing. P‐values were calculated using the Mixed‐effects model with Tukey's multiple comparisons test, as both fixed (such as cell types and culture time) and random effects (such as batch effects and donor variability) exist in the experiments. Differences between experimental groups were considered as significant when ^*^ *p* < 0.0332, ^**^ *p* < 0.0021, ^***^ *p* < 0.0002, and ^****^ *p* < 0.0001, n ≥ 3.

In recent decades, immunotherapy and the treatment with CAR T cells in particular have become a central focus for engaging the immune system in the fight against cancer, producing remarkable preclinical and clinical progress.^[^ ^43^, ^44^, ^45^, ^46^ ^]^ To visualize and evaluate the infiltration and cytotoxic ability of the CAR T cells against solid tumors surrounded by physical barriers and FAP‐mediated immune suppression in vitro, we further design the therapeutic trials with dual targeting against PSCA and FAP antigens using our microphysiological tumor model. In the following, we compared the immunotherapeutic effect of a UniCAR T cell therapy system in 3D P+H spheroids in beads to that in 2D cell cultures (**Figure** **4**a). Under 2D conditions, T cells can immediately come into contact with cancer cells once added to the plate (Figure 4b). Enhanced T‐cell accumulation and aggregation were observed when targeting either PSCA, FAP, or both antigens after 20 h of culture (Figure S15, Supporting Information). In the alternative case of PEGDA bead models, activated T cells must first overcome physical and biochemical barriers via migration and infiltration through the complex matrix created by the elastic hydrogel matrix and FAP‐positive cells surrounding PC3 spheroids. We show in the following that these factors significantly impact the kinetics and the outcomes of the UniCAR T cell therapy. To assess the UniCAR T cells' migration and infiltration abilities, P+H (10:1) spheroids were selected due to their resemblance to both solid and metastatic tumors in terms of structure and distribution.^[^ ^47^ ^]^ To evaluate the influence of anti‐PSCA and anti‐FAP TMs in the cell culture medium, the migration statistics of UniCAR T cells in the presence of anti‐PSCA and anti‐FAP TMs (P+F), anti‐PSCA TMs (PSCA), anti‐FAP TMs (FAP), and without any TMs were compared during the first 60 min of the experiment. UniCAR T cell migration was tracked in the time‐lapse movies (Video S1, Supporting Information) on the well slide surface, within the gel, or on the surface of the spheroids (Figure 4c, see “T Cell Counting and Migration Tracking” for details). The migration statistics of wild‐type (WT) T cells were also calculated and used as a control. Interestingly, the displacement distribution images (Figure 4c(I); Figure S10, Supporting Information) show that the highest numbers of UniCAR T cells gather around hydrogel beads when both anti‐PSCA and anti‐FAP TMs are present. To quantify the differences in cell numbers, T cells were counted in a ring area with an inner diameter of 450 µm and an outer diameter of 800 µm (Figure 4d; Figure S11, Supporting Information). The number of T cells in the ring area reached a peak after ≈30 min. In the P+F group, there were ≈250 more UniCAR T cells on average compared to the starting time point, indicating a higher accumulation trend compared to other groups. In addition, the recorded T cell velocities (Figure 4c(II); Figure S12, Supporting Information) indicated the alternating periods of motility and arrest phases of T cells, in accordance with previous reports.^[^ ^48^ ^]^ We observed a trend suggesting lower average velocities of T cells in FAP and P+F groups in comparison with those in WT and PSCA groups (Figure S13, Supporting Information). The intensified accumulation and lower velocities of T cells in FAP and P+F groups could be attributed to the presence of HT1080 hFAP cells and sFAP in hydrogel beads,^[^ ^49^, ^50^ ^]^ which may synergistically attract UniCAR T cells upon the addition of anti‐FAP TMs. The lowest accumulation of UniCAR T cells in the PSCA group might be due to the short incubation time (0–60 min), additional immunosuppressive mechanisms,^[^ ^51^ ^]^ and the co‐influence of the velocity and antigenic stimulation.^[^ ^52^ ^]^

UniCAR T cells infiltrated into PEGDA hydrogel beads and reached the spheroids (Figure 4e,f) after ≈1 h. The cells explored the surface of spheroids during the next few hours (Video S2, Supporting Information). After ≈3 h, distinct regions of T cell accumulation were observed on the surfaces of P+H spheroids cultured with anti‐PSCA and anti‐FAP TMs (Figure 4f(II), while no clear accumulation regions were detected for other groups; 3D T cell distribution (green) on different spheroids in the P+H (10:1) beads was observed even after culturing for 24 h (Figure S14, Supporting Information). Coordinated cell gathering at a defined location suggests that the accumulation of UniCAR T cells on the P+H spheroids is not only improved by the dual targeting of PSCA and FAP but also enhanced by cell‐to‐cell signaling, causing a positive feedback loop. In the following sections, we explore the capability of T cells to effectively eliminate cancer cells.

### 2.4 Better Prediction of Immunotherapeutic Efficacy

**Figure 5:**
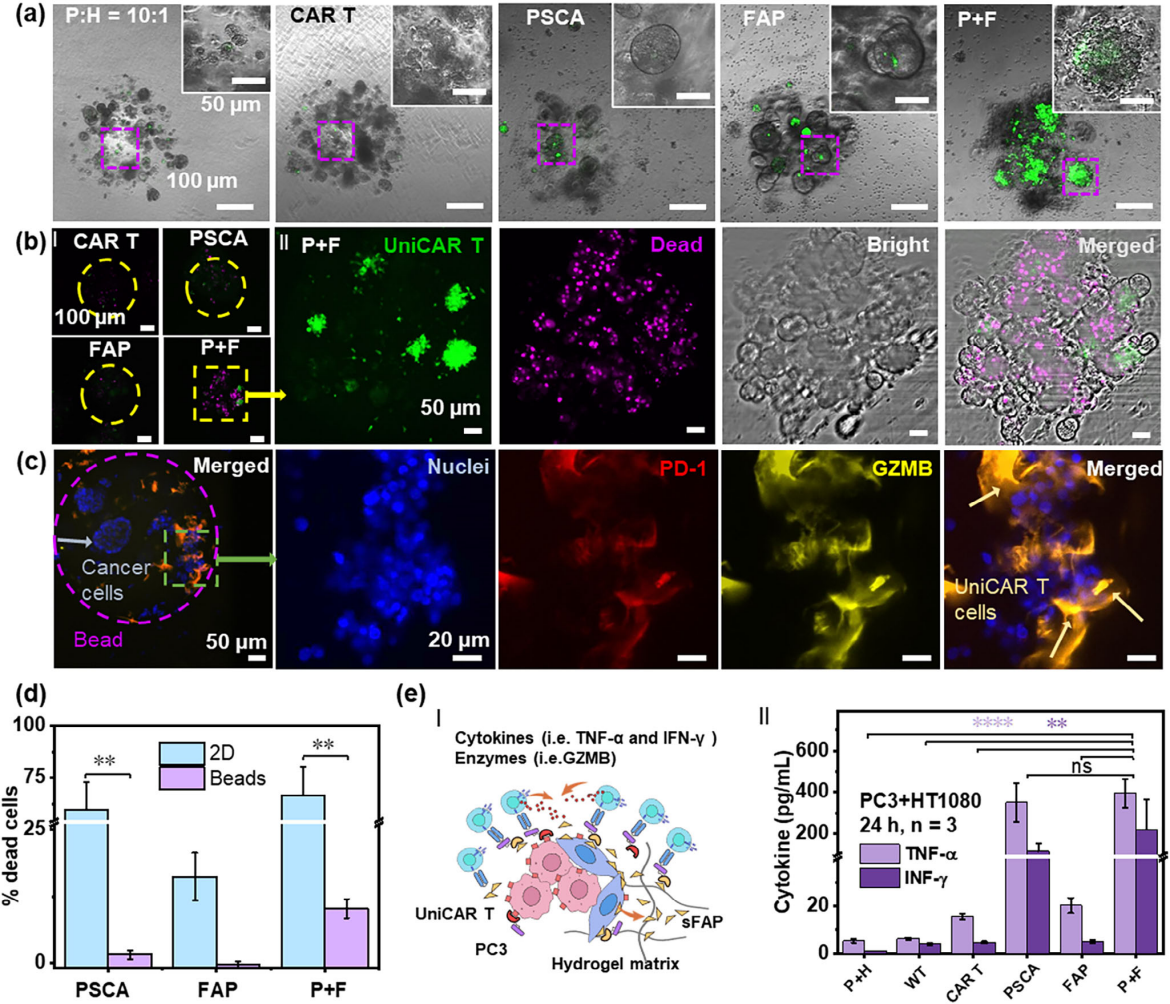
Therapeutic efficacy of T cells against P+H (10:1) spheroids and in 2D conditions. a) Representative accumulation of UniCAR T cells (green) on P+H (10:1) spheroids in hydrogel beads after 48 h of culturing under different conditions. b) Representative images of dead cells (magenta) staining treated by UniCAR T cells (green), after 48 h of culturing under different conditions. c) Representative immunostaining of T cell markers (blue: nuclei; red: PD‐1; yellow: GZMB). d) Differences in cytotoxicity between various P:H = 10:1 spheroids and 2D monolayer cultures. *P*‐values were calculated using the Two‐way ANOVA combined with Šídák's multiple comparisons test. e) (I) Illustration of possible scenarios for UniCAR T cells in PEGDA hydrogel beads; (II) Concentrations of TNF‐α and IFN‐γ in the supernatant obtained after 24 h of culturing under different conditions. P+H: only P+H spheroids; WT: P+H spheroids with wild‐type T cells; CAR T: P+H spheroids with UniCAR T cells; PSCA: P+H spheroids with UniCAR T cells and anti‐PSCA TMs; FAP: P+H spheroids with UniCAR T cells and anti‐FAP TMs; P+F: P+H spheroids with UniCAR T cells, anti‐PSCA TMs, and anti‐FAP TMs. *P*‐values were calculated using the One‐way ANOVA combined with Tukey's multiple comparison test. Differences between experimental groups were considered significant when ^*^ *p* < 0.0332, ^**^ *p* < 0.0021, ^***^ *p* < 0.0002, and ^****^ *p* < 0.0001, *n* ≥ 3.

To analyze whether the PEGDA multi‐spheroid models can better predict the outcome of CAR T therapy concerning treatment efficacy and timing, we assessed both monoculture and co‐culture conditions in 2D cultures and PEGDA beads. In the 2D cell cultures without physical barriers, ≈75% of cancer cells under both monoculture and co‐culture conditions were effectively killed within ≈20 h following the addition of UniCAR T cells and specific cancer‐targeting TMs, despite the presence of HT1080 hFAP cells (**Figure** **5**d; Figure S16, and Table S4, Supporting Information). A different situation was observed in the 3D PEGDA bead model, where effective T cell accumulation (green) and cell killing (magenta) occurred only when P+H (10:1) spheroids were cultured with both PSCA and FAP TMs after ≈48 h, indicating a synergistic cell‐killing effect under the dual‐targeting conditions (Figure 5a,b). In addition, immunostaining confirmed the presence of UniCAR T cells within hydrogel beads. These UniCAR T cells produced granzyme B (GZMB) to kill tumor cells. PD‐1 expression suggested an inhibitory mechanism that may downregulate T cell activity, potentially impacting overall treatment efficacy (Figure 5c). The suppressive cell‐killing effect associated with the ratio of FAP‐expressing cells was observed only in the PEGDA models (Figures S17–S20, Supporting Information). With the increased fraction of the FAP in the tumor microenvironment, reduced cytokine production was detected, indicating a lower interaction rate between T cells and cancer cells (Figure 4e; Figure S20, Supporting Information). These results align with an in vivo test in a previous report,^[^ ^53^ ^]^ illustrating the influence of FAP on signaling pathways, e.g., the ones controlled by nuclear factor‐κB, which are relevant to cancer metastasis and cytokine‐induced apoptosis.^[^ ^49^, ^54^ ^]^

## 3 Discussion

To date, CAR T cell therapy demonstrates relatively low efficacy in the clinical treatment of solid tumors.^[^ ^55^ ^]^ The scientific community believes that one of the main causes is the immunosuppressive role of the tumor microenvironment, composed of the extracellular matrix (ECM) and stromal cells, e.g., CAFs and mesenchymal stromal cells.^[^ ^32^, ^56^ ^]^ To improve therapeutic strategies, we need a deeper understanding of cancer progression and new insights into “unlocking the tumor microenvironment” through the bioengineering of tumor tissues, while accounting for the TME's influence. FAP, being overexpressed on CAF cells and present in soluble form (sFAP) in the extracellular matrix, is one of the crucial elements of TME that contributes to the remodeling of the ECM and degradation of the links within the tumor tissue, thereby simplifying its potential metastatic activity.^[^ ^29^, ^30^, ^31^ ^]^

In this study, we first investigated both physical and biochemical cues within hydrogel microbeads, focusing on the impact of FAP on tuning the microenvironment to influence the formation of microspheroids, thereby better mimicking in vivo conditions. The successfully prepared PC3‐PSCA/HT1080 hFAP 3D multi‐spheroid tumor models comprise tumor cells, an artificial supporting matrix, and FAP‐providing cells, which can be cultured for ≈20 days. Although HT1080 cells are different from fibroblasts, they share some similar properties, including their interaction with the extracellular matrix and migration behavior, and can be used as FAP suppliers.^[^ ^57^, ^58^ ^]^ PC3‐PSCA and HT1080 hFAP cells were co‐cultured in different ratios within PEGDA hydrogel beads, created through microfluidic droplet generation and rapid UV polymerization. System parameters for the formation of PEGDA hydrogel beads, like UV intensity, monomer and photoinitiator concentrations, and flow rates, can be fine‐tuned to adjust the important physical properties of the cell culture matrices, such as the elastic modulus and permeability. The PEGDA hydrogel beads of significantly lower elastic modulus than the prostate gland can support the growth and efficient proliferation of prostate cancer spheroids. Soft beads exhibit an elastic modulus in the range of some major organs, such as the lung and liver ^[^ ^6^ ^]^, which are also potential metastatic targets for testing the proliferation differences of the cells.^[^ ^59^ ^]^ The PEGDA hydrogel synthesis can be tuned to reach a relatively high elastic modulus of ≈10 MPa,^[^ ^60^ ^]^ indicating its potential for testing cell behavior in a wide range of elastic moduli (10^2^–10^7^ Pa). Through the chemical modification of the PEGDA polymer matrix,^[^ ^15^ ^]^ the model system can be further extended to study the influence of more complex extracellular conditions. The beads can also be used to test the survival and growth potential of other tumor cells, such as those in the pancreas and liver, which is an ongoing topic in our research.

Interestingly, we observed a reduction in size for the co‐cultured PC3‐PSCA and HT1080 hFAP spheroids (≈50–100 µm) compared to the PC3‐PSCA mono‐cultured spheroids (≈300 µm). This may be attributed to the effect that sFAP has on the degradation of extracellular matrix components, such as collagen.^[^ ^24^ ^]^ The morphological differences between spheroids, due to varying ratios of PC3‐PSCA and HT1080 FAP cells, may impact the stiffness of the 3D model, potentially affecting the efficacy of UniCAR T cell therapy. The stiffness can be tested and quantified using atomic force microscopy or Brillouin microscopy,^[^ ^61^, ^62^ ^]^ which is also a part of our ongoing research. Immunohistochemistry staining confirmed the presence and distribution of the FAP in the matrix around the PC3‐PSCA cells, depending on the co‐culture ratio of the PC3‐PSCA to HT1080 hFAP cells. Comparison to the clinical samples indicated that the obtained multi‐spheroid models closely resemble the initial or later stages of metastatic tumors in terms of tumor structures and FAP expression.^[^ ^47^ ^]^ Extended culturing of mouse prostate metastasis cell lines with or without Aldh1a3 gene knockdown, followed by comparison with mouse metastasis models, demonstrated that our in vitro model closely mirrors in vivo conditions, providing a valuable platform for further investigation into the mechanisms of prostate cancer metastasis.

Finally, the 3D multi‐spheroid tumor models were also utilized to evaluate the infiltration of UniCAR T cells into the hydrogel matrix to reach the tumor regions and the efficacy of immunotherapy implemented using the UniCAR approach. More realistic results were observed in the 3D multi‐spheroid models compared to traditional 2D culture. Therapeutic outcomes in both systems demonstrated the benefits of simultaneous dual targeting of the tumor marker PSCA and the tumor microenvironment marker FAP, as reflected in the increased efficacy of cancer cell killing. The coordinated migration behavior of UniCAR T cells resulted in the selective accumulation on the surface of tumor spheroids, thereby enhancing the killing efficacy. These findings also indicate that FAP acts as a physical or chemical shield for PSCA‐positive cells, yet it remains a promising target for cancer therapy,^[^ ^25^, ^63^ ^]^ despite the unclear mechanisms of sFAP formation.^[^ ^40^ ^]^ With an increased fraction of FAP‐expressing cells in the 3D multi‐spheroid model, reduced cell killing efficacy and cytokine production were observed, while cell killing remained effective under 2D culture conditions. These results indicate that the PEGDA 3D multi‐spheroid models are better at predicting the outcomes of cancer therapy. This system might be useful in adjusting the dosage of CAR T cells and TMs.

Looking ahead, although artificial models cannot be a general replacement for natural organisms in all contexts of cancer research, high‐throughput fabrication of reproducible miniaturized 3D cancer cell cultures still brings significant benefits. These models can open new avenues for reliable studies, accelerate research into complex cancer mechanisms, facilitate rapid ex vivo testing of therapies, and potentially reduce animal use. Further investigation into innovative material designs and integration of microfluidic systems can enable tunable bioreactors, allowing exploration of different facets of these systems, increasing their complexity, and broadening their potential applications. We are confident that this high‐throughput approach for constructing complex tumor models is indispensable for transitioning from using defined cell lines to patient‐derived samples, which can improve the representation of natural tumor heterogeneity, enhance the understanding of immunotherapeutic efficacy, and facilitate strategic planning of personalized therapies.

## 4 Experimental Section

### Materials

In this study, the following reagents were used without additional purification: poly(ethylene glycol) diacrylate (PEGDA, Mn 6000, Sigma–Aldrich), lithium phenyl‐2,4,6‐trimethylbenzoylphosphinate (LAP, Sigma–Aldrich), mineral oil (Sigma–Aldrich), Tween 20 (Sigma–Aldrich), phosphate‐buffered saline (PBS, Sigma–Aldrich), Dulbecco's phosphate‐buffered saline (DPBS, Sigma–Aldrich), sucrose (Carl Roth), gelatin (Carl Roth), calcein AM and propidium iodide (PI) (VWR (Corning)), hematoxylin (Sigma–Aldrich), eosin B (Sigma–Aldrich), ROTIMount (Carl Roth), Roticlear (Carl Roth).

### Preparation of PEGDA Hydrogel Beads and Size Measurement

PEGDA hydrogel beads were prepared at room temperature (20 °C) in a water‐in‐oil system using the in‐house built platform combining the T‐junction‐based microfluidic droplet generation with a setup for rapid UV polymerization, which was adapted from a previously reported droplet‐based real‐time monitoring system.^[^ ^64^, ^65^, ^66^ ^]^ The UV polymerization was carried out using a UV lamp (M365LP1, THORLABS) driven by constant electrical current sourced from a programmable DC power supply (SPD3303X‐E, SIGLENT). A cell culture medium (RPMI STINO, described in “Cell Culture”) supplemented with polymerization precursors (10% (w/v) PEGDA 6000 and 0.1% (w/v) LAP) was used as the water phase (WP). Mineral oil (Sigma–Aldrich) was used as the oil phase (OP). Liquids were stored in syringes and pumped into fluorinated ethylene propylene (FEP) tubing with an outside diameter of 1.6 mm and an inside diameter of 0.5 mm. The used flow rates were: q_WP_ = 5.5 µL min^−1^ and q_OP_ = 110 µL min^−1^. Droplets were first generated in the polytetrafluoroethylene (PTFE) T‐junction (inner diameter of 0.5 mm) and then quickly cross‐linked by localized exposure to UV light (mean intensity: 0.23–0.35 W cm^−2^; exposure time: ≈2.55 s) through the FEP tubing. The UV light intensities were measured using a UV intensity meter coupled with a 365‐nm sensor (diameter: 6.45 mm, Karl Suss, model 1000). The measured intensities correspond to the average intensities on the outside surface of the FEP tube. The experimental details and calculations related to UV irradiation are provided (Note SI and Figure S1, Supporting Information).

Sizes of PEGDA hydrogel beads were determined from wide‐field images obtained by an inverted microscope (Axio Observer, Zeiss). ImageJ analysis software was used to determine the particle size distribution. The distributions obtained from image analysis were fitted with a Gaussian distribution, giving the mean size and standard deviation (SD), as presented in the inset of Figure 1c. The polydispersity (*P*) expressed in percent was calculated by dividing the SD by the mean of the particle size distribution. The beads were considered monodisperse for *P* < 0.1.

### Physicochemical Characterization of PEGDA Hydrogel Beads

Freshly prepared PEGDA hydrogel beads were initially rinsed with PBS‐T (PBS containing 0.05% v/v Tween 20) three times. Following a single wash with DI water, the hydrogel beads were frozen at ‐20 °C overnight and then dried (Alpha 2–4 LSC plus, Christ) for 36 h. A scanning electron microscope (SEM, Phoenix XL) and a Fourier transform infrared spectrometer (FTIR, Perkin Elmer, Inc.) were used to characterize the microstructure and chemical composition, respectively, as reported in the previous study.^[^ ^13^ ^]^

### Elastic Modulus (Young's modulus) Test

Young's moduli of PEGDA hydrogel beads were measured on a MicroTester G2 system (CellScale) in DPBS at 37 °C. Briefly, randomly selected PEGDA hydrogel beads were placed into the test chamber filled with DPBS. A 1 mm‐by‐1 mm plate was affixed to one end of a tungsten beam with a diameter of 0.1524 mm. Parallel plate compression was performed, which subjected the beads to 25% compression at a controlled rate of 30 µm s^−1^. The recorded force‐displacement data were used to plot the stress–strain curves and calculate the Young's moduli (E) as follows:

(1) $$E(\mathrm{Pa})=\frac{\Delta \sigma }{\Delta \varepsilon }$$

where *σ* refers to stress and *ε* refers to strain. Stress and strain were calculated according to the following equations:

(2) $$\sigma =\frac{F\left(N\right)}{S\left(m^{2}\right)}$$

(3) $$\varepsilon =\frac{D\left(m\right)}{H\;\left(m\right)}$$

where *F* refers to force, *S* refers to the cross‐sectional surface area of hydrogel beads, *D* refers to tip displacement, and *H* refers to the original height of hydrogel beads.

### Cell Culture

The prostate cancer cell line PC3 and the fibrosarcoma cell line HT1080 were obtained from the American Type Culture Collection (ATCC). The PC3 overexpressing PSCA (PC3‐PSCA) and the HT1080 overexpressing human FAP (HT1080 hFAP) were prepared and confirmed by flow cytometry as described in previous reports.^[^ ^67^, ^68^ ^]^ PC3‐PSCA cells were cultured in RPMI STINO medium: Roswell Park Memorial Institute (RPMI 1640 ‐Gluta, Gibco) supplemented with 10% fetal bovine serum (FBS, Biochrom), 1% streptomycin and penicillin (Biochrom), 1% MEM non‐essential amino acid solution (Sigma–Aldrich), 1 mm sodium pyruvate solution (Sigma–Aldrich), and 2 mm Ala‐Gin (Sigma–Aldrich). HT1080 hFAP cells were cultured in DMEM STINO medium: Dulbecco's Modified Eagle Medium (DMEM + 4.5 g·L^−1^ D‐Glucose, – Sodium Pyruvate, Gibco) supplemented with 10% fetal bovine serum (FBS, Biochrom), and 1% MEM non‐essential amino acid solution (Sigma–Aldrich). The RM1 bone metastatic (BM) murine prostate carcinoma cell line, expressing GFP, was generously provided by Dr. Power (University of New South Wales, Australia) and was established as previously described.^[^ ^69^ ^]^ RM1(BM) cells were cultured in RPMI 1640 medium (Sigma–Aldrich) supplemented with 10% FBS, 2 mm L‐glutamine, 1% HEPES solution, 1 mm sodium pyruvate, 1% MEM non‐essential amino acid solution, 100 U mL^−1^ penicillin, 100 µg mL^−1^ streptomycin, and 4 µg mL^−1^ puromycin (all from Sigma–Aldrich). Cells were incubated under 5% CO_2_ at 37 °C. Cell culture medium was exchanged every 2–3 days. Cells were tested negative for Mycoplasma before experimentation.

### Incorporation of Cells into PEGDA Hydrogel Beads, Culturing, and Live/Dead Imaging

A solution of RPMI STINO medium containing 10% (w/v) PEGDA 6000, 0.1% (w/v) LAP, and predefined concentrations of cells was used as WP. Mono‐cultured spheroids were generated using PC3‐PSCA cells at a concentration of 1.2–1.5 × 10^7^ cells mL^−1^, HT1080 hFAP cells at a concentration of 3.0–3.2 × 10^7^ cells mL^−1^, and RM1 (BM) cells at a concentration of 2–2.5 × 10^7^ cells mL^−1^. For co‐cultured spheroids, a mixture of PC3‐PSCA and HT1080 hFAP cells with concentration ratios of 10:1 and 2:1 was utilized to form a total concentration of 1.2–1.5 × 10^7^ cells mL^−1^. Mineral oil was employed as OP. The flow rates for WP and OP were the same as the ones described in “Preparation of PEGDA Hydrogel Beads and Size Measurement”. The applied UV light intensity was 0.23 W cm^−2^. RPMI STINO medium was used to culture hydrogel beads containing cells, and the medium was refreshed every 2–3 days. Cell proliferation and spheroid growth were monitored using an optical microscope (Axio Observer, Zeiss). The average diameters of randomly selected spheroids were manually measured on micrographs using ImageJ (version: v1.54i). RM1 (BM) cells expressing GFP were imaged using a confocal fluorescence microscope (Olympus IX83) with an excitation wavelength of 488 nm and an emission detection range of 500–540 nm.

As for live/dead assay, spheroids in PEGDA hydrogel beads were firstly stained with calcein AM for 2 h (the first 10 days of culturing) or 3 h (after more than 10 days of culturing), and then with PI for 1 min. The solutions of staining chemicals were prepared following the instructions from the supplier (VWR (Corning)). Images were recorded using the confocal fluorescence microscope coupled to an excitation dichroic mirror (DM) (DM405/488/543/635), emission DM (DM560), and band‐pass filters (500–530 nm and 555–655 nm). Data visualization and quantification of cell viability were performed using a custom macro in ImageJ.

### Isolation and Genetic Modification of T Cells

Isolation of T cells and generation of UniCAR T cells were carried out as described in recent studies.^[^ ^67^, ^68^ ^]^ This study was approved by the local ethics committee of the Medical Faculty Carl Gustav Carus, Technical University Dresden (EK27022006). Briefly, T cells were isolated by using a human Pan T Cell Isolation Kit (Miltenyi Biotech GmbH, 130‐096‐535) from buffy coats provided by the German Red Cross (Dresden, Germany) after written consent of the voluntary donors. T cells without any modification were used as WT T cells. UniCAR T cells were generated by lentiviral transduction using a multiplicity of infection of 1–2. The proportion of UniCAR T cells was assessed via flow cytometry based on the co‐translated Enhanced Green Fluorescent Protein (EGFP) marker protein expression before each experiment.

### Production of TMs

The anti‐PSCA‐E5B9 and anti‐FAP‐E5B9 TMs were prepared as described previously.^[^ ^67^, ^68^ ^]^ Briefly, 3T3 cell lines expressing either anti‐PSCA‐E5B9 or anti‐FAP‐E5B9 TMs were produced by using lentiviral gene transfer. TMs were purified via His‐Tag using nickel‐nitrilotriacetic acid affinity chromatography (Qiagen GmbH). SDS‐PAGE (Sodium dodecyl‐sulfate polyacrylamide gel electrophoresis) and Western Blot were used to determine the concentration and purity of the TMs.

### Immunofluorescent and H&E Staining

To observe the 3D structure of PC3‐PSCA, HT1080 hFAP, PC3‐PSCA and HT1080 hFAP (P+H) spheroids, the nuclei, PSCA, and FAP were targeted with Hoechst 33 258 (ThermoFisher, 1:10), PSCA antibody (conjugated with Alexa Fluor 594, BIOSSUSA, 1:100), and hFAP antibody (R&D, 1:100), respectively. A secondary antibody, Chicken anti‐Mouse IgG (H+L) (conjugated with Alexa Fluor 647, Thermo Fisher Scientific, 1:50) was used to target the hFAP antibody. To stain the spheroids directly in PEGDA hydrogel beads precultured for 7 days, the beads were transferred to 18 µ‐slides and washed three times with PBS. Afterward, the PBS containing 4% paraformaldehyde was used to fix the spheroids for 10 min. Following the fixation step, the beads were incubated for 5 min in 10 mm EDTA to dissolve the gel matrix and release the spheroids. After permeabilization with 0.2% Triton X‐100 for 10 min, the spheroids were blocked using 3% BSA solution in PBS for 2 h. Primary antibodies (both anti‐PSCA and anti‐hFAP) were then added to incubate the spheroids at 4 °C overnight. After washing twice with tris‐buffered saline containing 0.1% Tween 20 (TBS‐T) and once with TBS, a secondary antibody was added and incubated for 1 h. Finally, the spheroids were incubated with Hoechst 33 258 for 10 min. After washing, Prolong (ThermoFisher) antifade reagent was added to prevent fluorescence quenching. Images were recorded using the confocal fluorescence microscope (Olympus IX83) coupled to the excitation DM (DM405/488/543/635), emission DM (DM490/640), and band‐pass filters (425–475 nm, 555–625 nm, and 655–755 nm). To compare extracellular matrix differences, collagen and fibronectin staining were performed. Collagen I (Collagen I polyclonal antibody, rabbit/IgG, Invitrogen, 1:100) and fibronectin (Fibronectin monoclonal antibody (FN‐3), mouse/IgG1, eBioscience, 1:100) were used as primary antibodies. As secondary antibodies, goat anti‐rabbit IgG conjugated with Alexa Fluor 647 and donkey anti‐mouse IgG (H+L) conjugated with Alexa Fluor 546 (both from Thermo Fisher Scientific, 1:200) were used.

To stain the sections of spheroids, PEGDA hydrogel beads containing spheroids were embedded into a mixed gel that contains 20% sucrose and 7.5% gelatin. Sections of 40 µm thickness were then created using a cryostat microtome under 22 °C (CM1850, Leica). After drying at 37 °C for 20 min, the sections were fixed with 4% paraformaldehyde for 10 min. The mixed gel can be washed off by using TBS‐T buffer at 50–60 °C for ≈10 min. The staining process for sections follows the same method as bead staining described previously, except for the primary antibody incubation parameters (2 h at room temperature). For H&E staining, the nucleus and cytoplasm were stained with hematoxylin and eosin B for 3 min and 30 s, respectively. Images were acquired using an Axio Imager A1 microscope (Zeiss).

### T Cell Counting and Migration Tracking

PEGDA hydrogel beads with cancer spheroids were first prepared and cultured in a 6‐well plate at 37 °C under 5% CO_2_ for 7 days. Then, for each tracking experiment, a single bead was transferred to 1 well of an 18‐well µ‐Slide (ibidi, uncoated). This step was followed by adding 50 µL of cell culture medium with WT T cells, UniCAR T cells, UniCAR T cells + anti‐PSCA‐E5B9 TMs, UniCAR T cells + anti‐FAP‐E5B9 TMs, or UniCAR T cells + anti‐PSCA‐E5B9 TMs + anti‐FAP‐E5B9 TMs, separately. Time‐lapse images were captured using an optical microscope (Axio Observer, Zeiss) and monitored in ZEN3.3 software. Images were taken every 30 s over a time span of 1 h. Experiments were replicated at least three times under the same conditions. A circular region of interest with a diameter of 450 µm was manually selected to track the T cells using ImageJ (version: v1.54i). Then, the TrackMate 7^[^ ^70^ ^]^ (ImageJ plugin) was used to track the T cells moving inside and outside of the circular region. T cells were counted within a ring area with an inner diameter of 450 µm and an outer diameter of 800 µm. Hessian detector and LAP tracker were selected to accurately identify T cell positions in the captured images. The detection threshold was manually adjusted depending on the individual images.

### T Cell Treatment Experiments and Killing Efficacy

PEGDA hydrogel beads with cancer spheroids were first prepared and cultured in a 6‐well plate at 37 °C under 5% CO_2_ for 7 days. Then the beads were transferred to 96‐well plates for T cell co‐culturing incubation. Five beads with PC3‐PSCA spheroids, HT1080 hFAP, or P+H spheroids were placed in each well. 200 µL of cell culture medium with WT T cells, UniCAR T cells, UniCAR T cells + anti‐PSCA‐E5B9 TMs, UniCAR T cells + anti‐FAP‐E5B9 TMs, or UniCAR T cells + anti‐PSCA‐E5B9 TMs + anti‐FAP‐E5B9 TMs was added to the wells. The concentration of T cells was 0.5 × 10^6^ cells·mL^−1^, and the concentration of TMs was 5 pmol·mL^−1^. Z‐stack images showing T cell (green) distribution within beads were captured after 24 and 48 h of culture.

Killing efficacy was determined using PI staining (as described in “Incorporation of Cells into PEGDA Hydrogel Beads, Culturing, and Live/Dead Imaging”). After staining, the spheroids were imaged with the confocal fluorescence microscope. For the determination of TNF‐α and INF‐γ concentrations, cell supernatants were collected for enzyme‐linked immunosorbent assay (ELISA) after co‐culturing for 24 h. The procedures outlined in the protocols provided with the ELISA kits purchased from Thermo Fisher Scientific Inc. were followed. Cell killing data was quantified by manually comparing the dead cell area to the spheroid area in Z‐stack images (interval = 20 µm) in ImageJ.

T cell immune response markers PD‐1 and GZMB were detected via immunostaining following the protocol for cell staining in hydrogel beads (refer to “Immunofluorescent and H&E Staining” for details). Recombinant PD‐1 antibody (conjugated with CF 488, antibodies‐online, 2 µg·mL^−1^) and GZMB antibody (conjugated with Alexa Fluor 555, antibodies‐online, 1:100) served as primary antibodies. Hoechst 33 258 served as the contrast agent. Images were recorded using the confocal fluorescence microscope (Olympus IX83) coupled to the excitation DM (DM405/488/543/635), emission DM (DM490/560), and band‐pass filters: 425–475 nm, 500–530 nm, and 555–625 nm.

### T Cell Treatment Experiments on a Cell Culture Plate

PC3‐PSCA and HT1080 hFAP cells with a ratio of 1:0, 10:1, 2:1, and 0:1 were seeded separately in a 96‐well plate. Each well had an initial cell number of 5 × 10^4^ cells. After overnight culturing at 37 °C under 5% CO_2_, 200 µL of cell culture medium with WT T cells, UniCAR T cells, UniCAR T cells + anti‐PSCA‐E5B9 TMs, UniCAR T cells + anti‐FAP‐E5B9 TMs, or UniCAR T cells + anti‐PSCA‐E5B9 TMs + anti‐FAP‐E5B9 TMs was added to the wells. The concentration of T cells was 0.5 × 10^6^ cells·mL^−1^, and the concentration of TMs was 5 pmol·mL^−1^. Cell culture medium without any T cells and TMs served as a control. Cell lysis was determined using calcein AM cell staining. Images were captured after 20 h of culture. Cell lysis was measured by comparing the residual area of cells covering the plate in each group to that of the control group. Calcein AM was used to stain the cells for 1 h to obtain the area of cell coverage. Images were recorded using the confocal fluorescence microscope (Olympus IX83) as described in “Incorporation of Cells into PEGDA Hydrogel Beads, Culturing, and Live/Dead Imaging”. Quantification of cell lysis was performed manually in ImageJ.

### Immunofluorescent Staining of Mouse Bone Marrow Metastases

The mouse experimental procedures were approved by the Medical Faculty of the Technische Universität Dresden and the Landesdirektion Sachsen, and were conducted under the same project referenced in 42.^[^ ^42^ ^]^ All data used in this study were obtained retrospectively from this prior research. No new animal experiments were performed. Sections of mouse leg bone were stained with GFP to visualize tumor nodules, endomucin for blood vessels, and DAPI for nuclei staining as a control. Additional details are provided (Notes SIII & SIV, Supporting Information).

### Clinical Specimens

Clinical samples were obtained from the University Clinic Münster. The tissue sample was part of the tissue bank of the Gerhard‐Domagk‐Institut für Pathologie, University Clinic Münster, Germany. Patients treated at the university hospital provided consent for the use of their data in research and education. No patient‐identifiable and clinical data were used for analysis, and the tissue was imaged retrospectively in an anonymized manner. The tissue sample was routinely processed by fixing in 4% formaldehyde overnight, drying, fixing in paraffin, and H&E staining.

### Statistical Analysis

Data preprocessing involved scaling by constant factors and the exclusion of outliers. Data were reported as mean ± SD. *p*‐values were calculated using the one/Two‐way ANOVA or Mixed‐effects model combined with Tukey's, Šídák's, or Dunnett's multiple comparison test or Fisher's LSD test, using GraphPad Prism 9, depending on the analyzed dataset. Differences between experimental groups were considered as significant when ^*^ *p* < 0.0332, ^**^ *p* < 0.0021, ^***^ *p* < 0.0002, and ^****^ *p* < 0.0001, *n* ≥ 3.

## Conflict of Interest

The authors declare no conflict of interest.

## Supporting information



Supporting Information

Supplemental Video 1

Supplemental Video 2

## Data Availability

The data that support the findings of this study are openly available in BioArxiv at https://doi.org/[doi], reference number 608227.
