# Supplementary material for: Microphysiological Solid Tumor Models in Hydrogel Beads for CAR T Cell Immunotherapy Evaluation
Source: Adv Sci (Weinh). 2025 Jul 24;12(41):e08267. doi: 10.1002/advs.202508267 (PMC12591166; doi:10.1002/advs.202508267)
Supplement: Supplementary file 1 — Supporting Information [file ADVS-12-e08267-s003.pdf]

Supporting Information

Table S1. Fibroblast activation protein (FAP) and its effects

Points	Explanations	Ref.
Existing area	FAP exists in intracellular and extracellular soluble, truncated forms, and is over-expressed by cancer-associated fibroblasts. As reported, it is expressed almost exclusively in pathological tissues, such as those affected by fibrosis, arthritis, and cancer.	[1]
Tumor progression	FAP can promote invasion of various cell lines, such as endothelial, melanoma, ovarian, and oral, by altering cell cycle and proliferation signal pathways.	[2]
Extracellular remodeling	FAP has been shown to degrade extracellular matrix components, such as fibrin and collagen.	[1, 3].
Immuno-suppressive	FAP can act as a tumor suppressor, abrogating tumorigenicity through regulation of cell growth and survival.	[4, 5]

SI Note: UV irradiation experiments and calculations

UV intensity. Due to the limited measurement range of the UV light sensor, we measured the intensities of emitted UV light when the lamp was driven by the DC electric current in the range from 0.05 to 0.55 A, and then calculated the linear fit to extrapolate the UV light intensities obtained under the current drive in the range from 1 to 1.5 A (defined as measured intensity, see Figure S1b). The illustration of the measurement setup geometry is shown in Figure S1c. The peak intensity (directly under the UV LED) and the mean intensity contributing to polymerization at the level of the FEP tube top surface were calculated by using MATLAB (R2022b) based on the measured intensity and the spatial radiation distribution data from the M365LP1-SpecSheet provided by the supplier (Table S2). The distance between the UV LED and the top surface of the FEP tube was measured using a digital micrometer (Schut).

Table S2. UV light intensity produced for different supplied electric currents.

Electric current (A)	Intensity ($W \cdot cm^{-2}$)		
	Measured	Peak	Mean
1.00	0.36	0.48	0.23
1.25	0.46	0.62	0.29
1.50	0.55	0.74	0.35

Exposure time calculation. The injection flow rates for cell culture medium and mineral oil were q_{WP} = 5.5 μL·min^{-1} and q_{OP} = 110 μL· min^{-1}, respectively. The total amount of liquid flowing through the tube per min (V_{min}) was 115.5 mm^3. The inside diameter of the tube is 0.5 mm, and thus, the inner cross-sectional surface area (S_{in}) is $0.25^2 \cdot \pi$ mm^2 ($\pi \approx 3.14$). The length of liquid flowing through the tube per minute (L_{min}) was V_{min}/S_{in} = 588.24 mm. The length of the tube placed within the irradiated area (L_{irr}) was 25 mm. Hence, the exposure time (equivalent to the time required for the liquid to pass through the irradiated area) can be calculated as $(L_{irr}/L_{min}) \cdot 60$ s ≈ 2.55 s.

SII Note: FTIR analysis

The band at 2888 cm^{-1} can be assigned to the asymmetric stretching of CH_2. The bands at 1726 cm^{-1} and 1098 cm^{-1} correspond to the symmetric stretching of C=O and stretching vibrations of C–O, respectively. The bands at 1645 cm^{-1}, 961 cm^{-1}, and 842 cm^{-1} are assigned to the vibrations of the aliphatic double bond C=C, out-of-plane symmetric stretching of CH_2=CH, and symmetric stretching of CH_2=CH, respectively.[6, 7]

SIII Note: Syngeneic immunocompetent tumor model

The syngeneic immunocompetent tumor model was established as reported[8]. Briefly, mouse prostate cancer bone metastatic RM1(BM) cells, with or without Aldh1a3 knockdown, were injected intracardially, followed by immunofluorescence analysis. Male C57BL/6 mice (8–9 weeks old) were anesthetized and placed in a supine position for tumor cell injection. Each mouse received 1 × 10^6 cells (shNS control or shAldh1a3) into the left ventricle. Three days post-injection, mice were euthanized via cervical dislocation. The legs were removed, and the knees were separated from the femur and tibia. The bones were then fixed in 4% paraformaldehyde (PFA) overnight at 4 °C. The next day, bones were washed in PBS, air-dried, and decalcified using 2 mL of Osteosoft reagent (Merck Millipore) at 37 °C for 5 days. After decalcification, the bones were embedded in OCT Tissue-Tek compound (Sakura) and sectioned into 10 μm-thick

slices using a cryotome. Bone sections were stored at -20 °C until further processing. All animal experiments were approved by the Landesdirektion Sachsen.

SIV Note: Immunofluorescence staining of bone metastases

Frozen bone sections were allowed to equilibrate at room temperature (RT) for 30 minutes before processing. The area of interest was marked using a Pap-Pen (Thermo Fisher Scientific). Sections were fixed with 4% PFA for 5 minutes, followed by washing and permeabilization with 0.01% Triton X-100 in PBS. After washing three times with PBS, sections were blocked using Protein Block Serum-Free (DAKO, Agilent) for 30–45 minutes at RT. Primary antibody (GFP and endomucin) incubation was performed overnight at 4 °C in DAKO Antibody Diluent (Agilent) containing background-reducing components. Then, sections were washed three times with PBS and incubated with the appropriate secondary antibody in DAKO Antibody Diluent for 1.5 hours at RT. Following secondary antibody incubation, sections were washed three times with PBS and stained with DAPI (1 mg mL^{-1}, 1:5000 in PBS) for 5 minutes at RT. Finally, slides were mounted using Fluorescent Mounting Medium (DAKO, Agilent) and stored at 4 °C overnight. Imaging was performed using the WF Slide Scanner Axioscan (Zeiss). A list of antibodies used in this study is provided in Supplementary Table S3.

Table S3. Antibodies used in the immunofluorescence staining of bone metastases.

Antibody	Source	Dilution	Source
GFP Tag	Rabbit	1:200	Thermo Fisher Scientific, #A6455
Endomucin	Goat	1:200	Thermo Fisher Scientific, #PA5-47648
Anti-Rabbit IgG (Alexa Fluor 488)	Donkey	1:350	Thermo Fisher Scientific, #A32731
Anti-Goat lgG (Alexa Fluor 555)	Donkey	1:350	Thermo Fisher Scientific, #A-21432

SV Note: PC3-PSCA cell culture in U-bottom plate, bulk Matrigel, and micro Matrigel beads

For 96-well U-bottom plate culturing, 200 µL of cell medium containing approximately

5000 or 70000 cells was added to each well. To prepare the Matrigel bulk, a 5 mg mL^{-1} Matrigel PBS solution with a concentration of 1.2–1.5×10^7 cells mL^{-1} PC3-PSCA cells was seeded on a petri dish (d = 60 mm) with 2 mL of added cell culture medium. For the preparation of Matrigel beads, a T-junction-based microfluidic setup, as described in the main text, was used without any UV gelation system (Figure S4). The flow rates were: q_{WP} = 5.5 µL min^{-1} and q_{OP} = 55 µL min^{-1}. Droplets were initially generated in the polytetrafluoroethylene (PTFE) T-junction (inner diameter of 0.5 mm) and stored in the fluorinated ethylene propylene (FEP) tubing for 10 min at room temperature before being transferred to a petri dish.

SVI Note: Cell lines

The prostate cancer cell line PC3 (RRID: CVCL_0035) and the fibrosarcoma cell line HT1080 (RRID: CVCL_0317) were obtained from the American Type Culture Collection (ATCC). The prostate cancer cell line PC3 was genetically modified via lentiviral transduction following previously published protocols to express PSCA and named PC3-PSCA.[9] The fibrosarcoma cell line HT1080 was genetically modified to express human FAP (hFAP) and named HT1080 hFAP.[10] The RM1 bone metastatic (BM) murine prostate carcinoma cell line, expressing GFP, was generously provided by Dr. Power (University of New South Wales, Australia) and was established as previously described.[11] In brief, RM1 (RRID: CVCL_B459) cells were transfected with the pVITRO2 plasmid containing the GFP gene using Lipofectamine reagent and then were injected into C57BL/6 mice to perform migration to obtain the RM1(BM) cells. This subline of RM1 cells was used to establish a syngeneic murine model for bone prostate cancer metastasis in the native immune environment. RM1(BM) cells were then transfected with pLKO.1 puro vector constructs expressing shRNA against mouse Alhd1a3 or nonspecific control shRNA (shNS) following previously published protocols[8] and were named as RM1(BM) shAlhd1a3 and RM1(BM) shNS, respectively. This immunocompetent murine model with a high bone marrow metastatic burden allowed a fair comparison of the established *in vitro* and physiologically

relevant *in vivo* models.

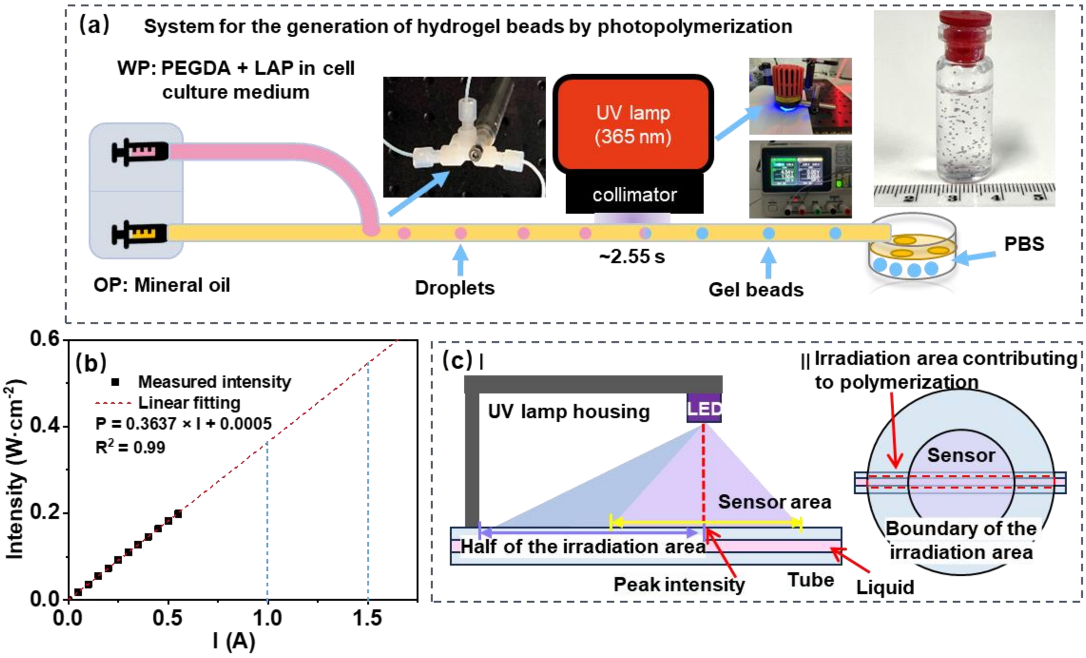

Figure S1. (a) Illustration and photographs of the PEGDA hydrogel beads generation platform. Macroscopic picture of produced hydrogel beads (stained with hematoxylin for visualization). (b) Measured UV intensity versus supplied direct current (DC) and the corresponding linear fit. (c) Illustration depicting the geometry of UV intensity measurements and the irradiated areas, shown from side view (I) and top view (II). WP: water phase; OP: oil phase.

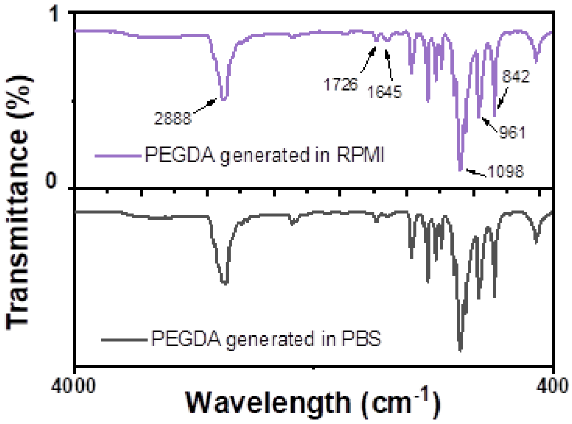

Figure S2. FTIR spectra of PEGDA hydrogel beads generated in RPMI-STINO cell culture medium and PBS.

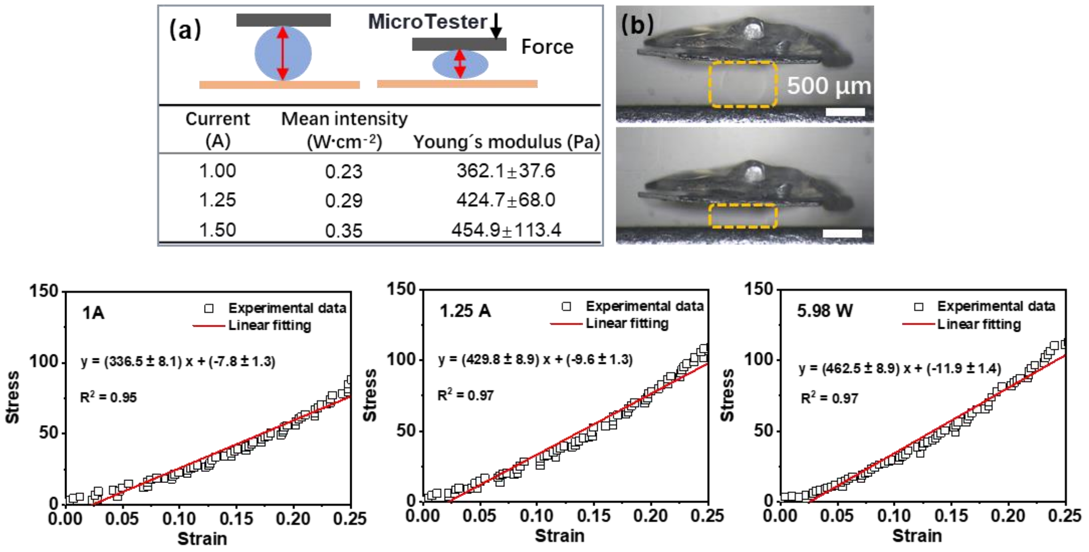

Figure S3. Elastic modulus of PEGDA hydrogel beads generated under different UV light intensities. (a) Illustration of the elastic modulus testing process and summary of elastic modulus for hydrogel beads prepared under different UV light intensities. (b) Representative images showcasing the testing process. (c) Representative stress-strain curves with corresponding linear fits used to determine the elastic modulus of PEGDA hydrogel beads (n = 5).

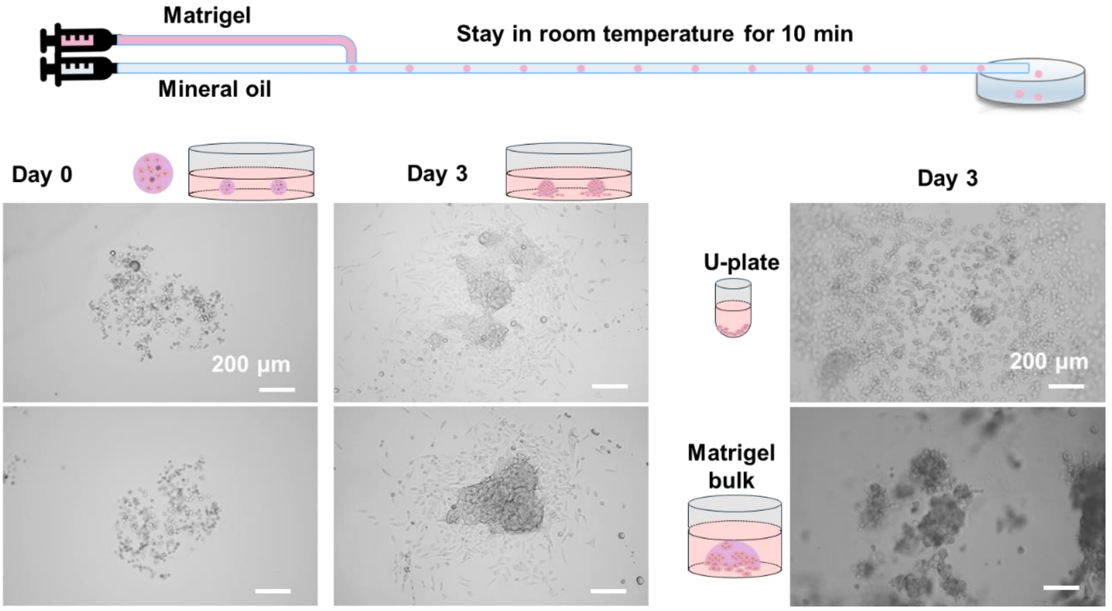

Figure S4. PC3-PSCA cell culture in U-bottom plate, bulk Matrigel, and micro Matrigel beads.

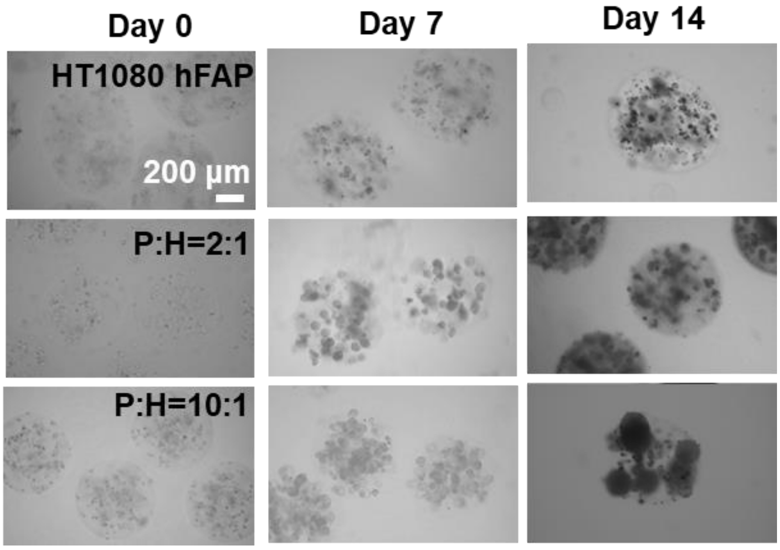

Figure S5. Representative optical micrographs illustrating the processes of spheroid formation and proliferation in PEGDA hydrogel beads loaded with HT1080 cells or PC3-PSCA (PC3) and HT1080 cells (P: H = 2:1 and 10:1).

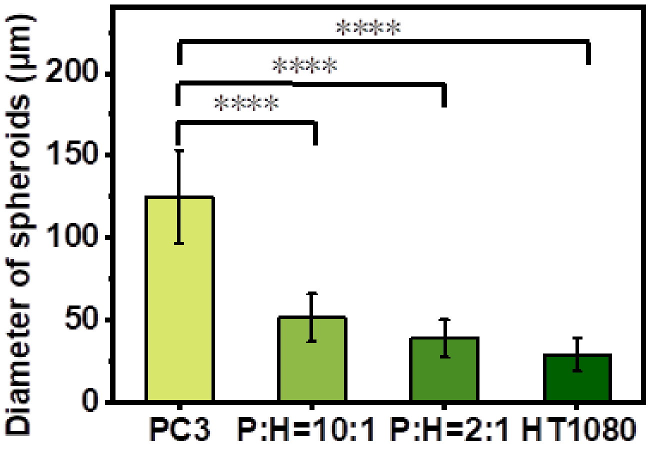

Figure S6. Average diameter of different spheroids measured on day 14, n = 100. p-values were calculated using the One-way ANOVA combined with Dunnett's multiple comparison test. Differences between experimental groups were considered as significant when *$p < 0.0332$, **$p < 0.0021$, ***$p < 0.0002$, and ****$p < 0.0001$, $n \geqslant 3$.

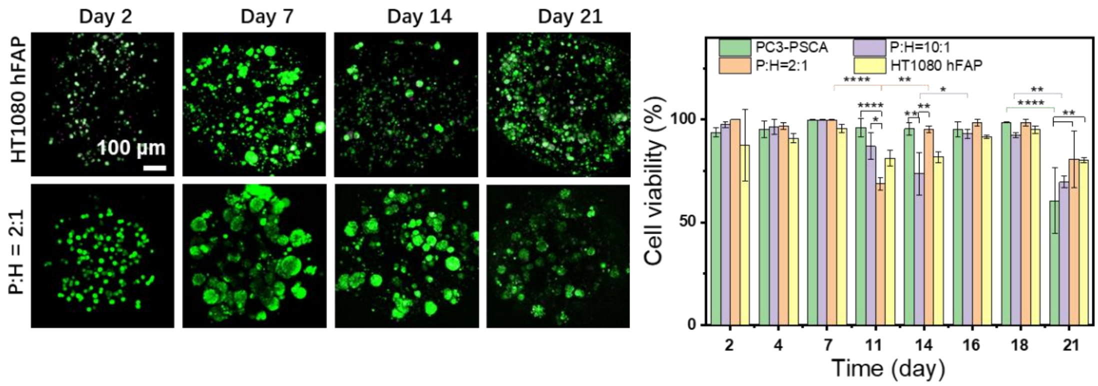

Figure S7. Representative fluorescent micrographs of live/dead (green/magenta) staining for P+H (2:1) and HT1080 hFAP spheroids and cell viability quantification in all groups. p-values were calculated using the Two-way ANOVA combined with Tukey's multiple comparison. Differences between experimental groups were considered as significant when $*p < 0.0332$, $**p < 0.0021$, $***p < 0.0002$, and $****p < 0.0001$, $n \geq 3$.

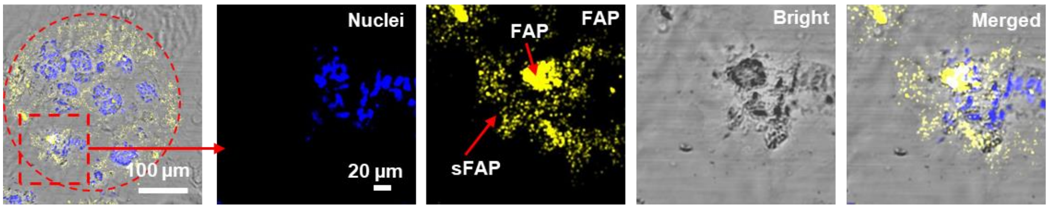

Figure S8. Immunostaining images of spheroid sections illustrating the distribution of FAP and sFAP.

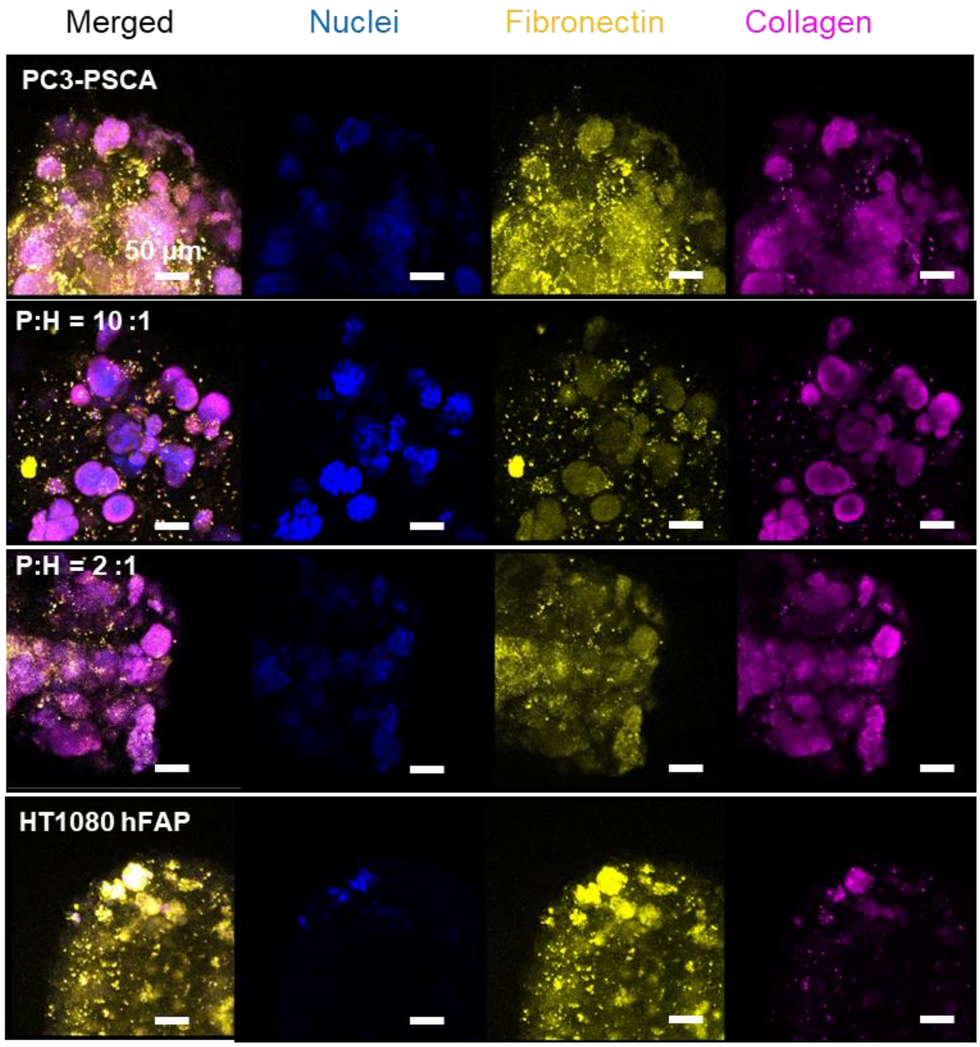

Figure S9. Immunostaining images of spheroid extracellular differences in PEGDA beads. Cyan: nuclei; yellow: fibronectin; magenta: collagen.

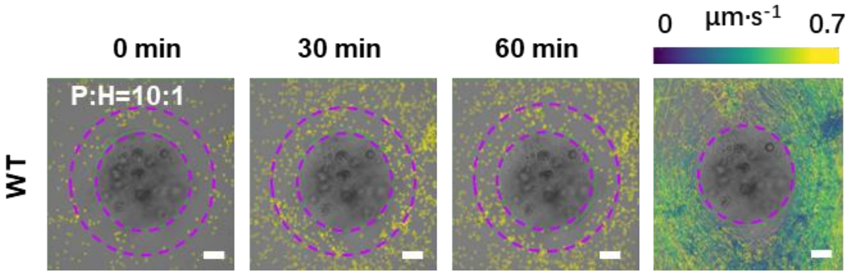

Figure S10. Representative images of wild-type (WT) T cell distribution and tracking of cell paths over 1 h.

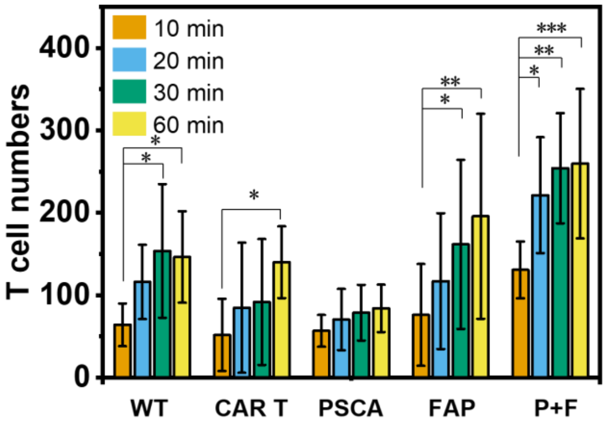

Figure S11. Average cell counts in ring areas designated in Figure 3a(I) after culturing the spheroids and T cells for a certain period; the ring area has an inner diameter of 450 μm and an outer diameter of 800 μm, n=3. WT: P+H spheroids with wild-type T cells; CAR-T: P+H spheroids with UniCAR-T cells; PSCA: P+H spheroids with UniCAR-T cells and anti-PSCA TMs; FAP: P+H spheroids with UniCAR-T cells and anti-FAP TMs; P+F: P+H spheroids with UniCAR-T cells, anti-PSCA TMs, and anti-FAP TMs. p-values were calculated using the Mixed-effects model with Tukey's multiple comparisons test, as both fixed (cell types and culture time) and random effects (such as batch effects and donor variability) exist in the experiments. Differences between experimental groups were considered as significant when *$p < 0.0332$, **$p < 0.0021$, ***$p < 0.0002$, and ****$p < 0.0001$, $n \geq 3$.

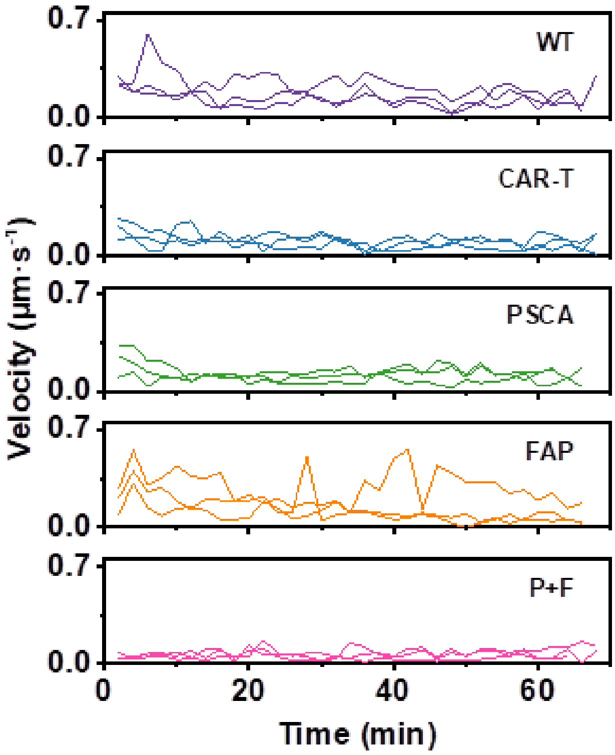

Figure S12. Representative velocities as a function of time for T cells under different microenvironmental conditions. **WT**: P+H (10:1) spheroids with wild-type T cells; **CAR T**: P+H spheroids with universal CAR T cells; **PSCA**: P+H spheroids with universal CAR T cells and anti-PSCA TMs; **FAP**: P+H spheroids with universal CAR T cells and anti-FAP TMs; **P+F**: P+H spheroids with universal CAR T cells, anti-PSCA TMs, and anti-FAP TMs.

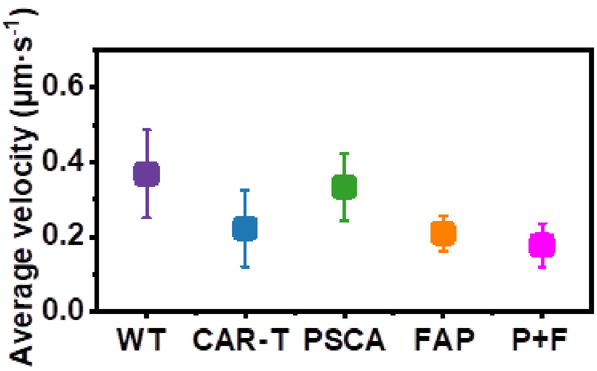

Figure S13. Average velocity of T-cell motion under different microenvironmental conditions calculated from Figure 3a(II). **WT**: P+H (10:1) spheroids with wild-type T cells; **CAR T**: P+H spheroids with universal CAR T cells; **PSCA**: P+H spheroids with

universal CAR T cells and anti-PSCA TMs; **FAP**: P+H spheroids with universal CAR T cells and anti-FAP TMs; **P+F**: P+H spheroids with universal CAR T cells, anti-PSCA TMs, and anti-FAP TMs.

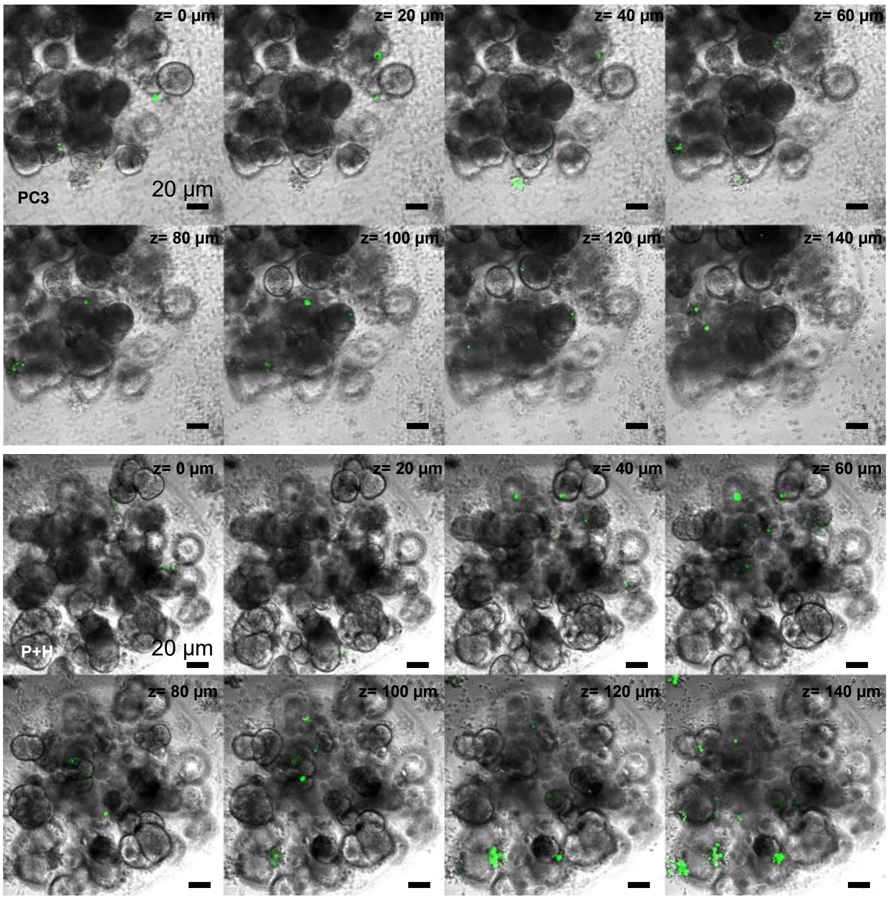

Figure S14. Representative images of T cell distribution (green) in 3D PC3 spheroids and P+H (10:1) spheroids after 24 h of culturing.

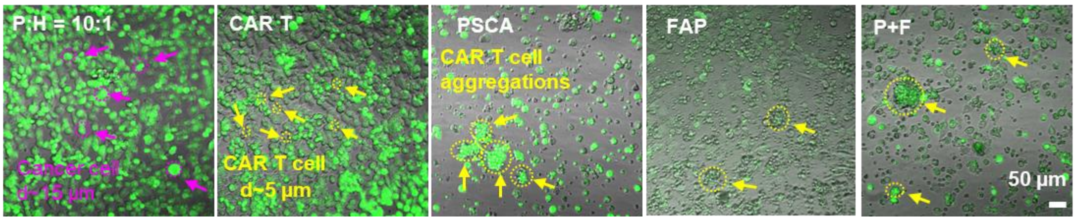

Figure S15. Representative examples of universal CAR T-cell accumulation around cancer cells under 2D culture conditions, in the presence of anti-PSCA and anti-FAP target modules (TMs) after 20 hours of culturing. The average diameter of a cancer cell is approximately 15 µm, while that of a T cell is about 5 µm, allowing for clear

differentiation under a microscope. Green: live cells. PC3-PSCA: only PC3-PSCA cells; CAR T: PC3-PSCA cells with universal CAR T cells; PSCA: PC3-PSCA cells with universal CAR T cells and anti-PSCA TMs; FAP: P+H cells with UniCAR-T cells and anti-FAP TMs; P+F: P+H cells with UniCAR-T cells, anti-PSCA TMs, and anti-FAP TMs.

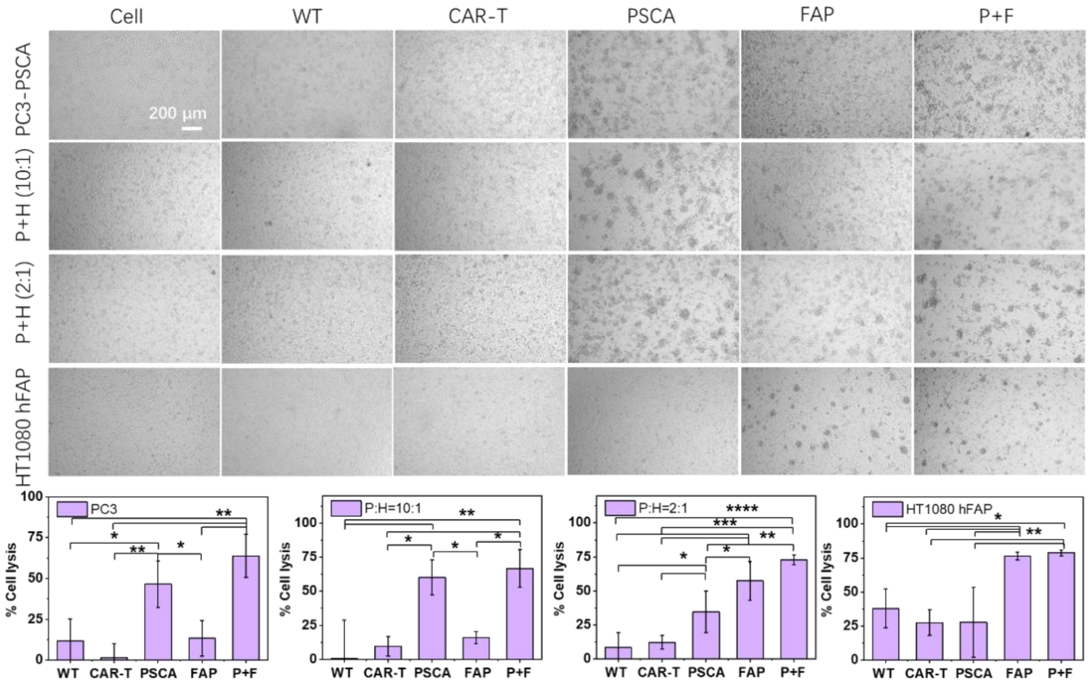

Figure S16. T cell cancer killing test in 2D culture. P+H (2:1 or 10:1): PC3-PSCA to HT1080 hFAP ratio = 2:1 or 10:1; Cell: without any T cells and TMs; WT: with wild-type T cells; CAR T: with universal CAR T cells; PSCA: with universal CAR T cells and anti-PSCA TMs. FAP: with universal CAR T cells and anti-FAP TMs; P+F: with universal CAR T cells, anti-PSCA TMs, and anti-FAP TMs. p-values were calculated using the Two-way ANOVA with Fisher's LSD test as limited test groups were used here. Differences between experimental groups were considered as significant when *p < 0.0332, **p < 0.0021, ***p < 0.0002, and ****p < 0.0001, n ≥ 3.

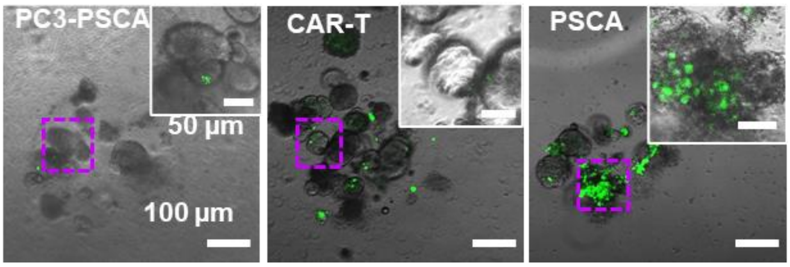

Figure S17. Universal CAR T cell (green) accumulation on PC3-PSCA spheroids after 48 h of culturing under different conditions. PC3-PSCA: only PC3-PSCA spheroids; CAR T: PC3-PSCA spheroids with universal CAR T cells; PSCA: PC3-PSCA spheroids with universal CAR T cells and anti-PSCA TMs.

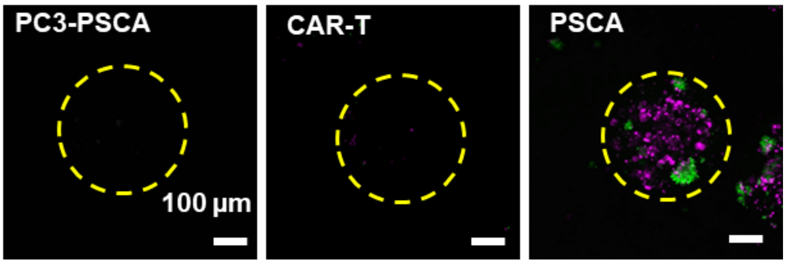

Figure S18. Dead cell (magenta) staining of PC3-PSCA spheroids after 48 h of culturing under different conditions. Green: universal CAR T cells. PC3-PSCA: only PC3-PSCA spheroids; CAR T: PC3-PSCA spheroids with universal CAR T cells; PSCA: PC3-PSCA spheroids with universal CAR T cells and anti-PSCA TMs.

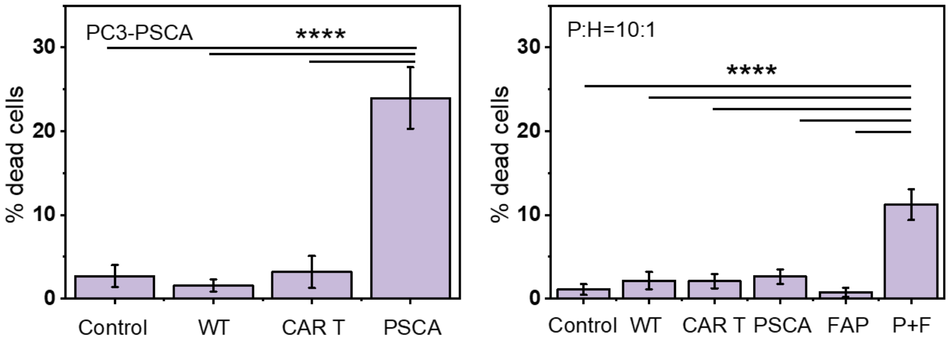

Figure S19. Tumor cell death rate in PC3-PSCA and P+H (10:1) 3D *in vitro* tumor models. p-values were calculated using the One-way ANOVA combined with Tukey's multiple comparison test. Differences between experimental groups were considered as

significant when *p < 0.0332, **p < 0.0021, ***p < 0.0002, and ****p < 0.0001, n ≥ 3.

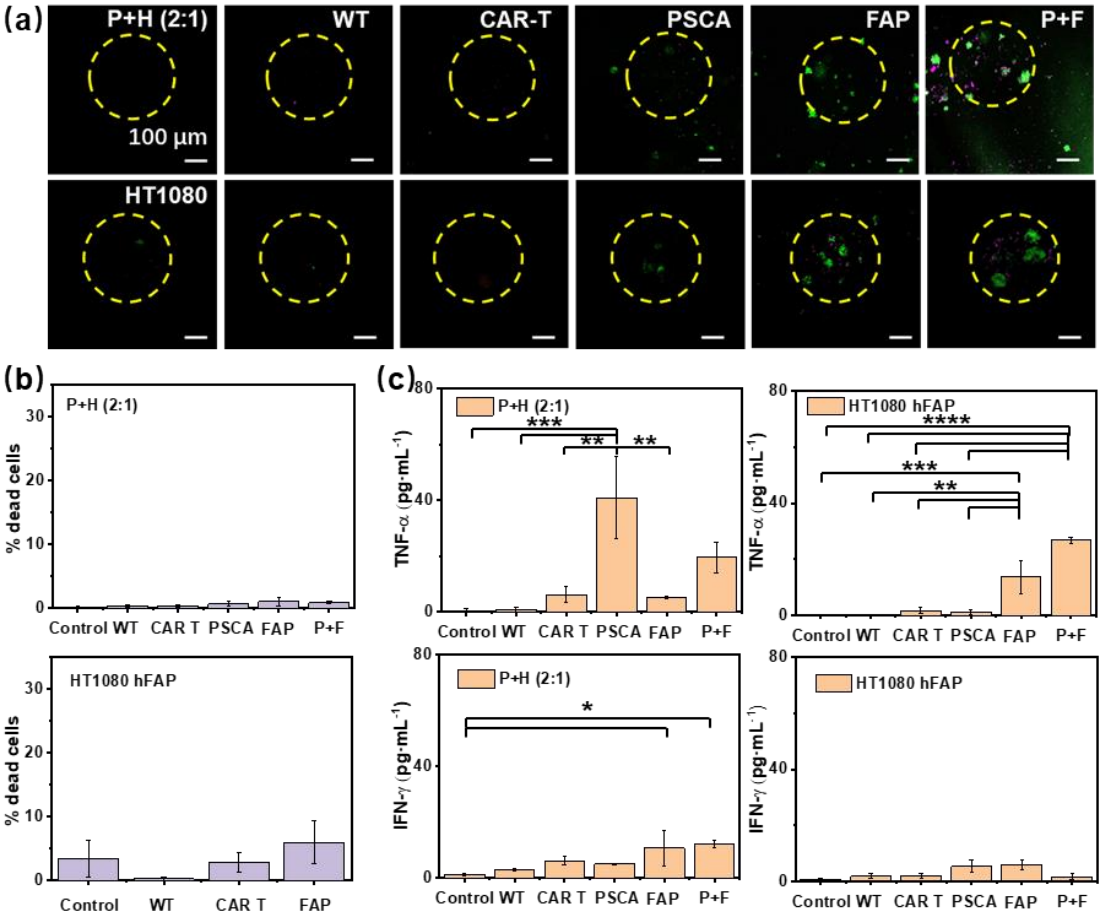

Figure S20. Tumor cell killing efficiency of universal CAR T cells in P+H (2:1) and HT1080 hFAP 3D *in vitro* tumor models. (a) Dead cell (magenta) staining of P+H (2:1) and HT1080 hFAP spheroids after 48 h of culturing. (b) Tumor cell death rate in P+H (2:1) and HT1080 hFAP 3D *in vitro* tumor models. (c) Concentrations of TNF-α and IFN-γ in the supernatant after 24 h of culturing under different conditions. Green: universal CAR T cells. PC3-PSCA: only spheroids; CAR T: spheroids with universal CAR T cells; PSCA: spheroids with universal CAR T cells and anti-PSCA TMs. FAP: spheroids with universal CAR T cells and anti-FAP TMs; P+F: spheroids with universal CAR T cells, anti-PSCA TMs, and anti-FAP TMs. p-values were calculated using the One-way ANOVA combined with Tukey's multiple comparison test. Differences between experimental groups were considered as significant when *p < 0.0332, **p <

0.0021, ***p < 0.0002, and ****p < 0.0001, n ≥ 3.

Table S4. Differences in dead cell ratio between 2D cultures and PEGDA beads under different cell culture conditions.

	**2D （after 20 h）**			**Beads（after 48 h）**		
Cell culture \ Target	PSCA	FAP	PSCA & FAP	PSCA	FAP	PSCA & FAP
PC3-PSCA	+	n/a	n/a	+	n/a	n/a
P+H (10:1)	+	-	+	-	-	+
P+H (2:1)	+	+	+	-	-	-
HT1080 hFAP	n/a	+	n/a	n/a	-	n/a

+ Significant cell death compared to CAR T group

- No significant cell death compared to CAR T group

n/a Not applicable

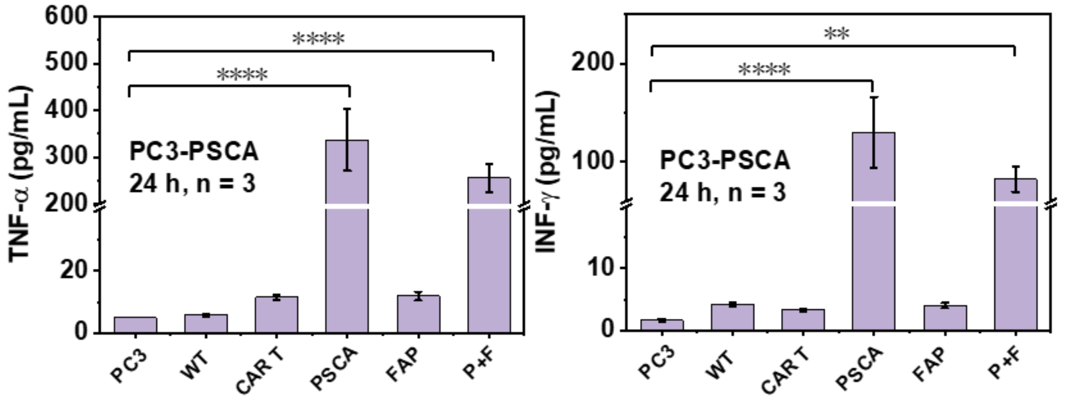

Figure S21. Concentrations of TNF-α and IFN-γ in the supernatant after 24 h of culturing under different conditions. PC3: only PC3-PSCA spheroids; WT: P+H spheroids with wild-type T cells; CAR T: PC3-PSCA spheroids with universal CAR T cells; PSCA: PC3-PSCA spheroids with universal CAR T cells and anti-PSCA TMs; FAP: PC3-PSCA spheroids with universal CAR T cells and anti-FAP TMs; P+F: PC3-PSCA spheroids with universal CAR T cells, anti-PSCA TMs, and anti-FAP TMs. p-values were calculated using the One-way ANOVA combined with Tukey's multiple comparison test. Differences between experimental groups were considered as

significant when *p < 0.0332, **p < 0.0021, ***p < 0.0002, and ****p < 0.0001, n ≥ 3.

References

[1] K. N. Lee, K. W. Jackson, V. J. Christiansen, C. S. Lee, J. G. Chun and P. A. McKee, Antiplasmin-cleaving enzyme is a soluble form of fibroblast activation protein, *Blood* **2006**, *107*, 1397-1404.
[2] A. A. Fitzgerald and L. M. Weiner, The role of fibroblast activation protein in health and malignancy, *Cancer Metastasis Rev* **2020**, *39*, 783-803.
[3] M.-H. Fan, Q. Zhu, H.-H. Li, H.-J. Ra, S. Majumdar, D. L. Gulick, J. A. Jerome, D. H. Madsen, M. Christofidou-Solomidou, D. W. Speicher, W. W. Bachovchin, C. Feghali-Bostwick and E. Puré, Fibroblast Activation Protein (FAP) Accelerates Collagen Degradation and Clearance from Lungs in Mice, *JBC* **2016**, *291*, 8070-8089.
[4] Z. Yuan, H. Hu, Y. Zhu, W. Zhang, Q. Fang, T. Qiao, T. Ma, M. Wang, R. Huang, Q. Tang, F. Gao, C. Zou, X. Gao, G. Wang and X. Wang, Colorectal cancer cell intrinsic fibroblast activation protein alpha binds to Enolase1 and activates NF-κB pathway to promote metastasis, *Cell Death Dis* **2021**, *12*, 543.
[5] T. Ramirez-Montagut, N. E. Blachere, E. V. Sviderskaya, D. C. Bennett, W. J. Rettig, P. Garin-Chesa and A. N. Houghton, FAPα, a surface peptidase expressed during wound healing, is a tumor suppressor, *Oncogene* **2004**, *23*, 5435-5446.
[6] L. Magalhaes, D. B. Andrade, R. D. S. Bezerra, A. I. S. Morais, F. C. Oliveira, M. S. Rizzo, E. C. Silva-Filho and A. O. Lobo, Nanocomposite hydrogel produced from PEGDA and laponite for bone regeneration, *J Funct Biomater* **2022**, *13*, 53.
[7] Y. Wu, L. Wang, B. Guo and X. M. P, Injectable biodegradable hydrogels and microgels based on methacrylated poly (ethylene glycol)-co-poly (glycerol sebacate) multi-block copolymers: synthesis, characterization, and cell encapsulation, *J Mater Chem B* **2014**, *2*, 3674-3685.
[8] I. Gorodetska, A. Offermann, J. Puschel, V. Lukiyanchuk, D. Gaete, A. Kurzyukova, V. Freytag, M. T. Haider, C. S. Fjeldbo, S. Di Gaetano, F. M. Schwarz, S. Patil, A. Borkowetz, H. H. H. Erb, A. Baniahmad, J. Mircetic, H. Lyng, S. Lock, A. Linge, T. Lange, F. Knopf, B. Wielockx, M. Krause, S. Perner and A. Dubrovska, ALDH1A1 drives prostate cancer metastases and radioresistance by interplay with AR-and RAR-dependent transcription, *Theranostics* **2024**, 14, 714-737.
[9] A. Feldmann, S. Stamova, C. C. Bippes, H. Bartsch, R. Wehner, M. Schmitz, A. Temme, M. Cartellieri and M. Bachmann, Retargeting of T cells to prostate stem cell antigen expressing tumor cells: comparison of different antibody formats, *Prostate* **2011**, 71, 998-1011.
[10] L. R. Loureiro, L. Hoffmann, C. Neuber, L. Rupp, C. Arndt, A. Kegler, M. Kubeil, C. E. Hagemeyer, H. Stephan, M. Schmitz, A. Feldmann and M. Bachmann, Immunotheranostic target modules for imaging and navigation of UniCAR T-cells to strike FAP-expressing cells and the tumor microenvironment, *J Exp Clin Cancer Res* **2023**, 42, 341.
[11] C. A. Power, H. Pwint, J. Chan, J. Cho, Y. Yu, W. Walsh and P. J. Russell, A novel model of bone-metastatic prostate cancer in immunocompetent mice, *Prostate* **2009**, 69, 1613-1623.
